# Prostaglandin E2 Signaling Mediates Oenocytoid Immune Cell Function and Lysis, Limiting Bacteria and *Plasmodium* Oocyst Survival in *Anopheles gambiae*

**DOI:** 10.3389/fimmu.2021.680020

**Published:** 2021-08-16

**Authors:** Hyeogsun Kwon, David R. Hall, Ryan C. Smith

**Affiliations:** Department of Entomology, Iowa State University, Ames, IA, United States

**Keywords:** PGE2 receptor, mosquito immunity, hemocytes, oenocytoid rupture, *Plasmodium*, prophenoloxidases, PGE2 signaling

## Abstract

Lipid-derived signaling molecules known as eicosanoids have integral roles in mediating immune and inflammatory processes across metazoans. This includes the function of prostaglandins and their cognate G protein-coupled receptors (GPCRs) to employ their immunological actions. In insects, prostaglandins have been implicated in the regulation of both cellular and humoral immune responses, yet in arthropods of medical importance, studies have been limited. Here, we describe a prostaglandin E2 receptor (*Ag*PGE2R) in the mosquito *Anopheles gambiae* and demonstrate that its expression is most abundant in oenocytoid immune cell populations. Through the administration of prostaglandin E2 (PGE2) and *AgPGE2R-*silencing, we demonstrate that prostaglandin E2 signaling regulates a subset of prophenoloxidases (PPOs) and antimicrobial peptides (AMPs) that are strongly expressed in populations of oenocytoids. We demonstrate that PGE2 signaling *via* the *Ag*PGE2R significantly limits both bacterial replication and *Plasmodium* oocyst survival. Additional experiments establish that PGE2 treatment increases phenoloxidase (PO) activity through the increased expression of *PPO1* and *PPO3*, genes essential to anti-*Plasmodium* immune responses that promote oocyst killing. We also provide evidence that the mechanisms of PGE2 signaling are concentration-dependent, where high concentrations of PGE2 promote oenocytoid lysis, negating the protective effects of lower concentrations of PGE2 on anti-*Plasmodium* immunity. Taken together, our results provide new insights into the role of PGE2 signaling on immune cell function and its contributions to mosquito innate immunity that promote pathogen killing.

## Introduction

Eicosanoids are lipid-derived signaling molecules that include prostaglandins (PGs), leukotrienes (LTs), lipoxins (LXAs), and epoxyeicosatrienoic acid (EETs), that serve important roles in immune regulation (1–3). Evidence suggests that these responses are evolutionally conserved across Metazoa, where eicosanoids significantly influence insect cellular immunity (4–10). In the mosquito, *Anopheles gambiae*, eicosanoids such as prostaglandin E2 (PGE2) and lipoxins have integral roles in mediating immune priming and mosquito susceptibility to malaria parasite infection (11, 12). However, our understanding of eicosanoid-mediated immune regulation in mosquitoes has remained incomplete due to the lack of characterized eicosanoid biosynthesis pathways and cognate receptors that are required to initiate eicosanoid signaling. As a result, there is a distinct need to identify the components that mediate eicosanoid signaling in mosquitoes and its respective contributions to innate immunity.

The recent characterization of a PGE2 receptor in the lepidopteran systems *Manduca sexta* (8) and *Spodoptera exigua* (10) have implicated PGE2 signaling *via* the PGE2 receptor in the activation of pathogen-associated molecular patterns (PAMPs) (8) and cellular immune function (10). While previous studies have linked PGE2 signaling to mosquito immunity (4, 11, 13), its cognate prostaglandin receptor has not yet been described in mosquitoes, leaving our understanding of prostaglandin signaling on immune function incomplete.

Based on *in silico* analyses of a recently described PGE2 receptor in a lepidopteran insect (8) and human prostanoid receptors, we identified a putative PGE2 receptor (*Ag*PGE2R) in *An. gambiae* orthologous to human PGE2-EP2 and EP4 receptors. Herein, we demonstrate that *Ag*PGE2R is predominantly expressed in oenocytoid immune cell populations and is integral to the production of antimicrobial peptides (AMPs) and a subset of prophenoloxidases (PPOs) that limit bacterial and *Plasmodium* survival in the mosquito host. Therefore, our study provides important new insights into mosquito PGE2 signaling, oenocytoid cell function, and the immune mechanisms that limit pathogens in the mosquito host.

## Materials and Methods

### Ethics Statement

The vertebrate animal protocols and procedures used in this study were approved by the Animal Care and Use Committee at Iowa State University (IACUC-18-228).

### Mosquito Rearing and *Plasmodium* Infection

*Anopheles gambiae* mosquitoes (Keele strain) were reared at 27°C and 80% relative humidity, with a 14/10-hour day/night cycle. Larvae were fed on fish flakes (Tetramin, Tetra), and adult mosquitoes were maintained on 10% sucrose solution. Female Swiss Webster mice were infected with a mCherry strain of *Plasmodium berghei* as previously described (14).

### Sequencing and Phylogenetic Analysis of the *Ag*PGE2 Receptor

A putative prostaglandin E2 receptor (AGAP001561) was identified in *An. gambiae* by BLAST P using orthologous sequence from the functionally characterized PGE2R in *M. sexta* (8). Cloning and sequencing of the full ORF cDNA of *Ag*PGE2R was performed from total RNA isolated from hemocytes perfused from non-blood fed female mosquitoes (n=50) using Direct-zol RNA Miniprep kit (Zymo Research). After DNase I treatment following the manufacturer’s protocol (New England Biolabs), 200 ng of total RNA was used for cDNA synthesis using the RevertAid First Strand cDNA Synthesis kit (Thermo Fisher Scientific). To obtain full length ORF cDNA (1463 bp), PCR was performed at 96°C for 2 min, followed by 40 cycles of 96°C 30s, 62°C for 60s, 72°C 60s, with a final extension at 72°C for 5 min using pge2R-F and pge2R-R primers listed in **Table S1**. Following gel purification of the PCR product, the amplified PCR product was cloned into pJET1.2/blunt Cloning Vector (Thermo Fisher Scientific) and sequenced by the Iowa State DNA Facility.

A phylogenetic tree was generated from amino acid sequences of human prostanoid and leukotriene receptors, and chemokine receptor 3 obtained from NCBI as previously described (8). Putative insect PGE2 receptors were predicted by protein BLAST search using PGE2 receptors sequenced from *M. sexta* and *An. gambiae*. The phylogenetic tree was produced with MEGA 7 software (15)

### *Ag*PGE2R Expression Analysis

Naïve and *P. berghei* infected mice were used for mosquito blood feeding and *Plasmodium* infection, respectively. To quantify relative abundance of receptor transcript level in the midgut and fat body, tissues were isolated from naïve (3-5 days old), 24 h blood-fed or 24 h *P. berghei* infected mosquitoes (n=40 per condition) in 1x sterile PBS. Hemolymph was separately perfused from mosquitoes (n=50) from similar conditions as described previously (14). Total RNA from isolated tissues was prepared using TRIzol (Thermo Fisher Scientific). Following hemolymph lysis in TRIzol reagent, total RNA was isolated using Direct-zol™RNA MiniPrep (Zymo Research). After DNase I treatment according to the manufacturer’s protocol (New England Biolabs), Total RNA from tissues (2 µg) and hemolymph (200 ng) was used for cDNA synthesis using the RevertAid First Strand cDNA Synthesis kit (Thermo Fisher Scientific). qRT-PCR was performed using PowerUp™SYBR^®^Green Master Mix (Thermo Fisher Scientific) with the ribosomal S7 protein transcript serving as an internal reference as previously (14). cDNA (1:5 dilution) amplification was performed with 500 nM of each specific primer pair using the following cycling conditions: 95°C for 10 min, 40 cycles with 95°C for 15 s and 65°C for 60 s. A comparative C_T_ (2^−ΔΔCt^) method was employed to evaluate relative transcript abundance for each transcript (16). A list of primers used for gene expression analyses are listed in **Table S1**.

### Western Blot Analysis

Western blot analysis was performed as previously described (14). Hemolymph was perfused from individual mosquitoes (n=35) at naïve, 24 h blood fed, or 24 h *P. berghei*-infected mosquitoes using incomplete buffer (anticoagulant solution without fetal bovine serum) containing a protease inhibitor cocktail (Sigma). Hemolymph protein concentrations were measured using Quick Start™Bradford Dye reagent (Bio-Rad). Protein samples (2 µg) were mixed with Bolt™LDS sampling buffer and sample reducing agent (Life Technologies), and heated at 70°C for 5 min before separation on 4-12% Bis-Tris Plus ready gel (Thermo Fisher Scientific). To determine PGE2R glycosylation in the hemolymph samples, protein samples were treated with PNGase F (Promega) according to the manufacturer’s instruction under denaturing conditions. Samples were resolved using Bolt™MES SDS running buffer (Thermo Fisher Scientific) for 90 min at 100 V. Proteins were transferred to PVDF membrane in Bolt™Transfer buffer (Life Technologies) for 1 h at 20 V, and then blocked in TBST buffer (10 mM Tris base, 140 mM NaCl, 0.05% Tween 20, pH 7.6) containing 5% non-fat milk for 1 hour at RT. For western blotting, the membrane was incubated with a 1:1000 dilution of rabbit anti-*Ag*PGE2R (Cys-NRSMSQTPKSSSFTDSNIIR: third intracellular loop) affinity purified antibodies (3.1 mg/ml; Pacific Immunology), pre-immune serum (1:1000), or rabbit anti-serpin3 (SRPN3) antibodies (1:1000) (14) in TBST blocking buffer overnight at 4°C. Membranes were washed three times for 5 min in TBST, then incubated with a secondary anti-rabbit alkaline phosphatase-conjugated antibody (1:7500, Thermo Fisher Scientific) for 2 h at RT. Following washing in TBST, the membrane was incubated with 1-Step™NBT/BCIP (Thermo Fisher Scientific) to enable colorimetric detection.

### Immunofluorescence Assays

Hemocyte immunofluorescence assays (IFAs) were performed as previously described (14). Hemolymph perfused from mosquitoes at naïve, 24 h post-blood meal and 24 h post-infection was placed on a multi-well glass slide (MP Biomedicals) and allowed to adhere at RT for 30 min. Cells were fixed with 4% paraformaldehyde for 15 minutes at RT, then washed three times in 1xPBS. Samples were incubated with blocking buffer (0.1% Triton X-100, 1% BSA in 1xPBS) for 24 h at 4°C and incubated with a 1:500 of rabbit anti-*Ag*PGE2R (CVRYRSATEPID: second extracellular loop) affinity-purified antibodies (0.6 mg/ml) (Pacific Immunology), or pre-immune serum (1:1000) in blocking buffer overnight at 4°C. After washing 3 times in 1xPBS, an Alexa Fluor 568 goat anti-rabbit IgG (1:500, Thermo Fisher Scientific) secondary antibody was added in blocking buffer for 2 h at RT. Slides were rinsed three times in 1xPBS and mounted with ProLong^®^Diamond Antifade mountant with DAPI (Life Technologies). Images were analyzed by fluorescence microscopy (Nikon Eclipse 50i, Nikon) and confocal microscopy (Leica SP5 X MP confocal/multiphoton microscope) at the Iowa State University Microscopy Facility.

### Phagocytic Cell Depletion Using Clodronate Liposomes

To validate that the *Ag*PGE2R is predominantly expressed on non-phagocytic oenocytoid immune cells, female mosquitoes were treated with either control liposome or clodronate liposome as previously described to deplete phagocytic immune cell populations (14, 17). Hemolymph was perfused from naive mosquitoes (n=40) at 24 h post-injection. Total RNA isolation, cDNA synthesis and qRT-PCR experiment were performed as described above. Relative abundance of *AgPGE2R* transcript level was determined between treatments.

### Endogenous PGE2 Titers

Mosquito samples for analysis of prostaglandin E2 (PGE2) were prepared as previously described (11). To determine how endogenous PGE2 level is regulated in the mosquito at different conditions (naïve, 24 h blood fed and 24 h *P. berghei* infection), hemolymph (5 µl per mosquito) was perfused from naïve, 24 h blood fed and 24 h *P. berghei* mosquitoes using the HBSS buffer, and a pool of hemolymph (50 µl) perfused from ten mosquitoes was used for measurement of PGE2 titer. PGE2 titer was measured by Prostaglandin E2 Monoclonal ELISA kit (Cayman Chemical) according to the manufacturer’s instructions. Absorbance was read at 412 nm using a microplate reader (Multi-mode reader, Biotek). PGE2 level was calculated using the Prostaglandin E2- Monoclonal program (4PL) at http://www.myassays.com (MyAssays Ltd., UK).

### Silencing *Ag*PGE2R by RNAi

dsRNA synthesis was performed as previously described (14). The N-terminus of *AgPGE2R* including the 5’ UTR and first 61 amino acid residues was selected for dsRNA synthesis. Specific primers listed in **Table S1** were designed to amplify a 421 bp DNA template for subsequent dsRNA production using cDNA synthesized from naïve whole female mosquitoes. The amplified PCR product was excised from an agarose gel, purified using Zymoclean Gel DNA Recovery Kit (Zymo Research), and cloned into a pJET1.2/blunt vector (Thermo Fisher Scientific). The plasmid DNA was amplified with T7 primers (**Table S1**) at 96°C for 2 min, followed by initial 10 cycles of 96°C 30s, 58°C for 60s, 72°C 60s, and subsequent 30 cycles of 96°C 30s, 72°C for 60s, 72°C 60s, with a final extension at 72°C for 5 min. MEGAscript RNAi kit (Thermo Fisher Scientific) was used for dsRNAs synthesis following the manufacturer’s instructions. dsRNA was precipitated with ethanol and resuspended in nuclease free water to 3 µg/µl. To determine the role of *Ag*PGE2R in *Plasmodium* development, mosquitoes (3-5 days old) were anesthetized on ice and intrathoracically injected with 69 nl (~200 ng) of dsRNA per mosquito using Nanoject III injector (Drummond Scientific Company). The dsRNA treated mosquitoes were kept at 19°C for 4 days, then the effects of gene silencing on expression of immune effectors, development of *P. berghei*, and clearance of *E. coli* infection were evaluated. To determine RNAi efficiency, mosquitoes (n=15) at 4 days post-injection and mosquitoes (n=15) at 24 h post-*P. berghei* infection were collected for total RNA isolation, cDNA synthesis and qRT-PCR analysis as described above.

To further validate RNAi efficiency on *Ag*PGE2R protein expression, western blot and IFA analyses were performed using hemolymph perfused from mosquitoes treated with dsPGE2R or dsGFP at 4 days post-injection. SDS-PAGE and Western blot analysis using protein samples (2 µg) were performed as described above. Relative quantification of protein bands from western blot scanning images were analyzed using ImageJ software (https://imagej.nih.gov/ij/). In addition, receptor silencing effect on the localization of *Ag*PGE2R was determined by IFA as described above.

### Effects of PGE2 Signaling on Gene Expression and PO Activity

To determine if PGE2 signaling *via Ag*PGE2R influences prophenoloxidase (PPO) and antimicrobial peptide (AMP) gene expression as previously described (18), we examined phenoloxidase activity and gene expression following PGE2 priming. Naïve mosquitoes were injected with 69 nl of either 500 nM or 2 µM PGE2, with 0.05% ethanol in 1xPBS as a control. At 24 h post-injection, mosquitoes (n=15 per treatment) were collected for RNA isolation, cDNA synthesis, and qRT-PCR analysis of gene expression (*PPO, lozenge,* and *AgPGE2R*), or were processed for IFA as described above. At 24 h post-injection of either PGE2 or 0.05% ethanol PBS, a pool of hemolymph perfused from mosquitoes (n=15, 10 µl per mosquito) in nuclease free water was prepared for analysis of phenoloxidase (PO) activity. The perfused hemolymph (10 µl) was mixed with 90 µl of 3, 4-Dihydroxy-L-phenylalanine (L-DOPA, 4 mg/ml) dissolved in nuclease free water as previously described (14). After initial 10 min incubation at room temperature, PO activity was measured at 490 nm every 5 min for 30 min, then the final activity was measured at 60 min using a microplate reader. Similar experiments were performed following *AgPGE2R*-silencing 4 days post-injection of dsRNA. PO activity was measured in gene-silenced mosquitoes injected with 500 nM PGE2 using perfused hemolymph from mosquitoes (n=15) at 24 h post-injection.

### *Ex Vivo* Oenocytoid Rupture Following PGE2 Application

Using filtered 1xPBS, hemolymph (~5 µl) was perfused from naïve mosquito (3-4 day old) and placed on a multi-well glass slide. To make a final concentration at 2 µM PGE2, the same volume of 4 µM PGE2 (~5 µl) was immediately applied to the hemolymph sample. As a control, 0.4% ethanol in 1xPBS (~5 µl) was applied to the hemolymph sample. After sealed with a coverslip, selected oenocytoids and granulocytes were video-recorded using NIS-Elements D software on a Nikon 50i microscope for one hour to capture live imaging of morphological changes of oenocytoids and granulocytes in response to 1xPBS or 2 µM PGE2. Videos were processed, cropped, and accelerated using Lightworks 2021.1. Still images were obtained from screenshots of the video using the Snipping Tool utility in Microsoft Windows. Similar experiments were performed in *GFP*- or *PGE2R*-silenced mosquitoes 4 days post-injection to evaluate the influence of *Ag*PGE2R on oenocytoid rupture following the application of 2 µM PGE2 as described above.

### PGE2 Priming on Bacterial Challenge

Bacterial challenge experiments were performed with slight modification from previous studies (19). Naïve female mosquitoes (3-5 day old) were intrathoracically injected with 69 nl of PGE2 (500 nM) or 0.05% ethanol PBS using a Nanoject III (Drummond Scientific). Kanamycin resistant *E. coli* constructed by transformation with mCherry2-N1 plasmid was cultured in Luria Bertani (LB) broth containing kanamycin (50 µg/ml) overnight at 37°C. This *E. coli* suspension (OD_600_ = 0.4, 10^8^ cells) was spun down at 8000 rpm for 5 min and the bacterial pellet was washed twice in 1xPBS. At 24 h post-PGE2 application, mosquitoes were challenged *E*. *coli* (~6950 cells in 69 nl) and collected at 6 h and 24 h post-infection. Individual mosquitoes were homogenized in 1 ml of LB broth. Mosquito homogenates (100 µl) were spread onto LB-kanamycin (50 µg/ml) agar plates. Bacterial plates were incubated overnight at 37°C and colony-forming units (CFU) per plate were assessed to quantify the level of infection. Additional experiments were performed in control- and *Ag*PGE2R-silenced mosquitoes to determine the role of *Ag*PGE2R in antimicrobial immunity was performed by similar *E. coli* challenge experiments as described above.

### Contributions of PGE2 and *Ag*PGE2R to *Plasmodium* Survival

Naïve mosquitoes (3-5 days old) were injected with either 69 nl of 500 nM PGE2 or 0.05% ethanol PBS. At 24 h post-injection, mosquitoes were challenged with *P. berghei* infection and kept at 19°C. Oocyst survival was assessed at 2 days and 8 days post-infection. To evaluate effect of silencing *Ag*PGE2R on parasite development, dsRNA treated mosquitoes were challenged with *P. berghei* and kept at 19°C until assessment of oocyst survival at either 2 days or 8 days post-infection. To determine whether anti-*Plasmodium* immunity mediated by PGE2 priming requires *Ag*PGE2R activation, mosquitoes treated with either dsPGE2R or dsGFP at 4 days post-injection were primed with PGE2 (500 nM) before challenging with *P. berghei* infection and oocyst survival was assessed at 8 days post-infection.

### Effect of Prophenoloxidases and Antimicrobial Peptides on *P. berghei* Infection

Due to the effect of the PGE2 signaling system on regulation of a set of PPOs and antimicrobial peptide genes, RNAi experiments were carried out with selected genes: *PPO1* (AGAP002825), *PPO7* (AGAP004980), *PPO8* (AGAP004976) and *CEC1* (AGAP000693). T7 primers were designed using the E-RNAi web application (http://www.dkfz.de/signaling/e-rnai3/idseq.php) and listed in **Table S1**. dsRNA synthesis was performed as described above. The effects of gene silencing were measured 2 days post-injection in whole mosquitoes (n=15) by qRT-PCR as described above. Mosquitoes were challenged with *P. berghei* at 2 days post-injection of dsRNA, and oocyst survival was assessed at 7 days post-infection. The influence of PPOs in limiting oocyst survival was further examined in mosquitoes co-silenced with *PPO1* and *PPO3* at 2 days post-injection of dsRNA were primed with PGE2 (500 nM) and challenged with *P. berghei* infection. Oocyst survival was assessed at 7 days post-infection.

## Results

### Characterization of the *Ag*PGE2 Receptor

Based on the previously described prostaglandin E2 receptor from the tobacco hornworm, *Manduca sexta* (8), we identified a candidate PGE2 receptor (*Ag*PGE2R; AGAP001561) in *An. gambiae*. Based on this annotation, a full-length transcript consisting of 1463 bp was isolated from perfused naïve *An. gambiae* hemolymph (**Figure S1**), encoding a predicted 404 amino acid protein (~46 kDa). *In silico* analysis suggests that the *Ag*PGE2R contains seven transmembrane domains and belongs to the rhodopsin-like family of G protein-coupled receptors (GPCRs; **Figures S1**, **S2**). Moreover, four N-glycosylated and six phosphorylation sites are predicted at the respective N- and C- terminus (**Figures S1**, **S2A**), suggesting that the *Ag*PGE2R undergoes post-translational modifications as in mammalian systems (20, 21). Phylogenetic analysis reveals a unique clade of insect receptors where *Ag*PGE2R is most similar to other putative dipteran PGE2 receptors, and contains core residues characteristic of other Family A GPCRs and human prostanoid receptors (**Figure S2**). These analyses also suggest that insect PGE2Rs are more closely related to human prostanoid receptors than human leukotriene receptors (CYSLTR1/2) (**Figure S2**), which also serve as important receptors for eicosanoid signaling.

**Figure 1:**
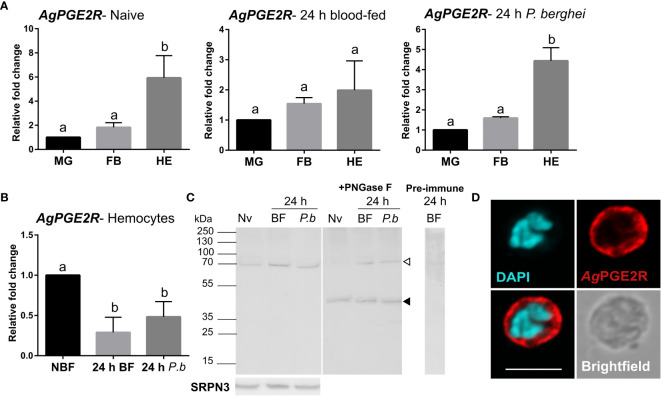
Characterization of a prostaglandin E2 receptor (PGE2R) in *Anopheles gambiae*. **(A)** *AgPGE2R* gene expression was examined by qRT-PCR in midgut (MG), fat body (FB), and perfused hemocytes (HE) under naïve (NV), blood-fed (24 h BF), or *P. berghei*-infected (24 h *P.b*) conditions. **(B)** *AgPGE2R* expression was more closely examined in hemocytes and compared across physiological conditions. For both **(A, B)**, letters indicate statistically significant differences (*P* < 0.05) when analyzed by a one-way ANOVA followed by a Tukey’s multiple comparison test using GraphPad Prism 7.0. Bars represent the mean ± SE of either three or four independent biological replicates. **(C)** Perfused hemolymph from naïve (NV), blood-fed (BF), or *P. berghei* infected (*P.b*) mosquitoes were examined by Western blot analysis. Specific bands were detected corresponding to a glycosylated *Ag*PGE2R product (bands at ~70 kDa, open arrowhead), or in which the receptor underwent deglycosylation with PNGase F treatment (~46 kDa, black arrowhead). The *Ag*PGE2R was detected using a rabbit antibody (1:1000) directed against intracellular loop 3 (ICL3). No bands were detected in a 24 h BF hemolymph sample incubated with pre-immune serum. Serpin 3 (SRPN3) was used as a protein loading control. **(D)** Immunofluorescence assays were performed on perfused hemocytes using a rabbit polyclonal antibody (1:500) against extracellular loop 2 (ECL2), revealing that PGE2R is localized to oenocytoid immune cell populations (scale bar, 5 µm).

To more closely characterize the role of the putative mosquito prostaglandin receptor, we examined *AgPGE2R* expression in the midgut, fat body, and hemocytes from different physiological conditions (naïve, 24 h blood-fed, and 24 h *P. berghei-*infected; **Figure 1A**). Under both naïve conditions and following *P. berghei* infection, receptor expression is highly enriched in hemocytes compared to other tissues (**Figure 1A**). However, when *AgPGE2R* expression was examined in tissues under different feeding conditions, only hemocytes displayed significant differences in *AgPGE2R* expression (**Figures 1B** and **S3**). Western blot analysis using mosquito hemolymph samples result in a single band of approximately 70 kDa (**Figure 1C**), much higher than the expected ~46 kDa MW of the annotated *Ag*PGE2R. As a result, we examined *Ag*PGE2R following PNGase F treatment to enzymatically remove protein glycosylation sites that result in the identification of a ~46 kDa *Ag*PGE2R protein matching the expected size of the annotated protein sequence (**Figure 1C**), indicating that *Ag*PGE2R undergoes substantial post-translational modifications through glycosylation. Following the incubation of pre-immune serum, no specific bands were detected (**Figure 1C**). Immunofluorescence assays revealed that *Ag*PGE2R is predominantly expressed in oenocytoid immune cell populations (**Figures 1D** and **S4**), although a weak signal is also detected in a small subset of granulocytes (**Figure S4A**). This is further supported by a recent scRNA-seq study of mosquito hemocytes, which demonstrate that *AgPGE2R* expression is highest in oenocytoid populations while only weakly expressed in specific granulocyte subtypes (22) (**Figure S5**). We also further validate *AgPGE2R* localization to oenocytoids through the use of clodronate liposomes (CLD) to ablate phagocytic granulocyte populations as previously described (14, 17). qRT-PCR analysis demonstrates that *Ag*PGE2R transcript remains unchanged in perfused hemocyte samples from mosquitoes treated with CLD, while the expression of *eater* [a known phagocytic marker (22–24)] was significantly reduced (**Figure S4B**). Together, these results provide strong support that *Ag*PGE2R is strongly expressed in oenocytoid immune cell populations.

### PGE2 Biosynthesis and Influence on Mosquito Immune Function

**Figure 2:**
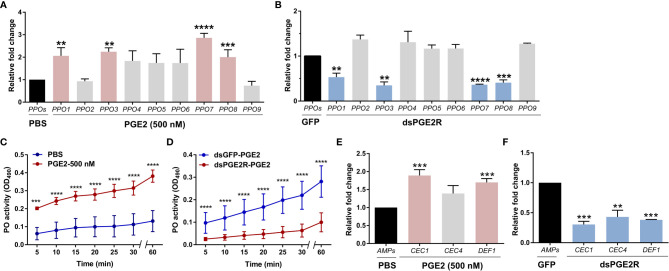
PGE2 signaling influences phenoloxidase (PO) activity and antimicrobial peptide (AMP) gene expression. Following treatment with PGE2 (500nM) **(A)** or when *AgPGE2R* was silenced **(B)**, the expression of all nine mosquito prophenoloxidases (PPOs) were examined by qRT-PCR and compared to respective PBS or GFP controls. For both **(A, B)**, mosquitoes were pooled (n=15) for analysis. Data were analyzed using an unpaired t test to determine differences in relative gene expression for each respective PPO gene between treatments. Bars represent mean ± SE of three independent biological replicates. **(C)** PO activity was measured from perfused hemolymph in mosquitoes primed with PGE2 (500 nM) and compared to PBS controls 24 h post-treatment (n=15 per treatment). **(D)** Additional experiments were performed measuring PO activity in which PGE2 (500nM) was injected into *AgPGE2R*- or *GFP*-silenced (control) mosquitoes (n=15 per treatment). For both **(C, D)**, measurements (OD490) were taken for DOPA conversion assays at 5-min intervals from 0 to 30 min, as well as a final readout at 60 min. Data were analyzed using a two-way repeated-measures ANOVA followed by Sidak’s multiple comparisons using GraphPad Prism 7.0. Bars represent mean ± SE of 3 independent experiments. In addition, PGE2 priming induced expression of antimicrobial peptide (AMP) genes **(E)**, while *AgPGE2R-silencing* reduced AMP expression **(F)**. For **(E, F)**, data were analyzed using an unpaired *t*-test to determine differences in relative gene expression of each respective AMP gene between treatments. Bars represent mean ± SE of three independent replications. For all data, asterisks denote significance (**P < 0.01, ***P < 0.001, ****P < 0.0001). Genes that display significant differences in gene expression following PGE2 treatment are shaded in pink, while those genes differently regulated by *AgPGE2R*-silencing are displayed in blue.

To understand the molecular mechanisms of PGE2 signaling in *An. gambiae*, PGE2 titers were measured from perfused hemolymph under naïve, 24 h blood-fed, or 24 h *P. berghei-*infected conditions. Similar to previous reports (11), PGE2 levels only reached measurable levels in the hemolymph following *P. berghei* infection as a result of ookinete invasion (**Figure S6**) (11). Based on our earlier observations of *Ag*PGE2R localization in oenocytoid cell populations (**Figures 1D** and **S4**), we explored what role PGE2 signaling may have on prophenoloxidase (PPO) expression. qRT-PCR analysis demonstrates that a subset of PPOs which includes *PPO1*, *PPO3*, *PPO7* and *PPO8* were upregulated in response to PGE2 treatment (**Figures 2A** and **S7**), which corresponds to those PPOs most enriched in oenocytoid populations (22) (**Figure S5**). In contrast, when *AgPGE2R* is silenced *via* RNAi (**Figure S8**), the same subset of *PPOs* (**Figure 2B**) are significantly downregulated, suggesting that the expression of *PPO1*, *PPO3*, *PPO7* and *PPO8* is regulated by PGE2 signaling. Since lozenge has been previously implicated in the expression of these same subset of PPOs (22), we examined the potential that *lozenge* may be influenced by PGE2 signaling (**Figure S9**). Following PGE2 treatment (500 nM) or *Ag*PGE2R-silencing, *lozenge* did not display significant differences in expression (**Figure S9**), suggesting that *lozenge* expression is independent of PGE2 signaling.

To determine if these PGE2-mediated effects on PPO expression also influence phenoloxidase (PO) activity, we performed dopa conversion assays following PGE2 treatment. Compared to PBS controls, PGE2 treatment significantly increased hemolymph PO activity (**Figure 2C**). Moreover, PGE2 treatment in *Ag*PGE2R-silenced mosquitoes resulted in substantially less PO activity than GFP-silenced controls (**Figure 2D**), providing additional support that PGE2 signaling promotes PO activity *via* the *Ag*PGE2R.

Previous studies have also implicated PGE2 signaling on the synthesis of antimicrobial peptides (AMPs) in *Anopheles albimanus* (4). Therefore, we examined the expression of major AMPs that display strong expression in *Anopheles* oenocytoid cell populations (22). Following PGE2 treatment or *Ag*PGE2R-silencing as in **Figures 2A, B** examining *PPO* expression, PGE2 treatment significantly increased the expression of *cecropin 1* (*CEC1*) and *defensin 1* (*DEF1*) (**Figure 2E**), while the silencing of *Ag*PGE2R reduced *CEC1*, *CEC4* and *DEF1* (**Figure 2F**). Together, these data support that PGE2 signaling *via Ag*PGE2R is integral to phenoloxidase and AMP signaling pathways in the mosquito immune response.

### PGE2 Signaling Promotes Antimicrobial Activity

Based on the influence of PGE2 signaling on AMP expression (**Figure 2E**), we examined the antimicrobial effects mediated *via* PGE2 by challenging mosquitoes with *E. coli* following PGE2 treatment. At 6 h post-challenge, PGE2-treated mosquitoes displayed significantly less bacteria when compared to PBS controls (**Figure S10A**). We identified a similar trend at 24 h post-challenge, yet these results were not significant (**Figure S10A**). Moreover, we demonstrate that *Ag*PGE2R is essential to these responses, where the effects of PGE2 treatment are abrogated following *AgPGE2R*-silencing (**Figure S10B**), providing further support that PGE2 signaling limits bacterial growth in the mosquito host.

### PGE2 Signaling Mediates Anti-*Plasmodium* Immunity

**Figure 3:**
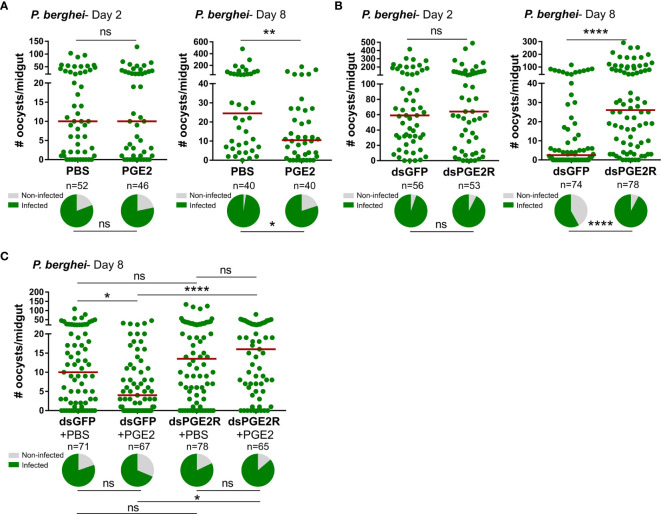
PGE2 signaling mediates *An*. *gambiae* anti-*Plasmodium* immunity. Following priming with PGE2 (500 nM), *P. berghei* infection was measured by evaluating oocyst numbers at 2 days and 8 days post-infection **(A)** and compared to PBS controls. When *AgPGE2R*-silenced mosquitoes were challenged with *P. berghei*, early oocyst numbers (Day 2) were comparable to GFP controls, yet displayed significant differences at Day 8 **(B)**, reminiscent of previously described late-phase immunity phenotypes. When PGE2 was administered to *GFP-* and *AgPGE2R*-silenced mosquitoes, PGE2 priming significantly limited oocyst survival in control GFP mosquitoes, while there were no effects of PGE2 priming on oocyst survival in *AgPGE2R*-silenced background **(C)**. All infection data were analyzed by a Mann–Whitney test using GraphPad Prism 7.0. Median oocyst numbers from three or more independent biological replicates are indicated by the horizontal red line. The prevalence of infection (% infected/total) is depicted for mosquitoes under each experimental condition and examined by X^2^ analysis to determine significance. Asterisks denote significance (*P < 0.05, **P < 0.01, ****P < 0.0001); ns, not significant. n = number of individual mosquitoes examined.

In agreement with the previously reported effects of PGE2 priming on anti-*Plasmodium* immunity (11), we confirm that mosquitoes treated with PGE2 have significantly reduced oocyst numbers eight days post-infection (**Figure 3A**). However, when we more closely examine the timing of parasite killing as previously (14, 25–29), PGE2 treatment has no effect on the success of ookinete invasion when early oocyst numbers were assessed at two days post-infection (**Figure 3A**). This argues that PGE2 signaling influences oocyst survival similar to other known mediators of mosquito late-phase immunity (14, 25–29). To confirm the involvement of *Ag*PGE2R in mediating these responses, *AgPGE2R* was silenced *via* RNAi (**Figure S8**) and evaluated for its influence on *Plasmodium* survival. Similar to PGE2 treatment (**Figure 3A**), *AgPGE2R*-silencing did not influence early oocyst numbers at two days post-infection (**Figure 3B**). However, when oocyst survival was examined at eight days post-infection in the *AgPGE2R*-silenced background, there was a significant increase in oocyst numbers (**Figure 3B**). Together, these data independently support that PGE2 signaling is integral to *Plasmodium* oocyst survival, yet to further validate the role of *Ag*PGE2R in mediating the anti-*Plasmodium* effects of PGE2 signaling, we demonstrate that PGE2 priming requires *Ag*PGE2R function (**Figure 3C**).

**Figure 4:**
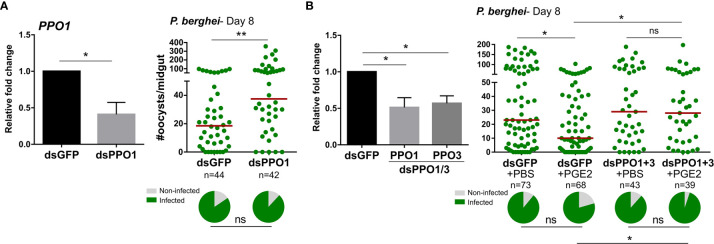
Anti-*Plasmodium* effects of PGE2 priming are mediated by mosquito prophenoloxidases (PPOs). Significant reduction of PPO1 gene expression enhanced oocyst survival at 7 days post-infection **(A)** *PPO1* gene expression was efficiently reduced following RNAi, resulting in a significant increase in *P. berghei* oocyst survival when evaluated 8 days post-infection. To verify the role of mosquito PPOs in mediating PGE2 immune activation, *PPO1* and *PPO3* were co-silenced and then primed with either PBS (control) or PGE2 **(B)**. Following challenge with *P. berghei*, oocyst survival was examined 7 days post-infection. Data were analyzed by an unpaired t test to determine RNAi efficiency and a Mann–Whitney test to assess oocyst survival using GraphPad Prism 7.0. Bars represent mean ± SE of three independent biological replicates. Median oocyst numbers from three or more independent biological replicates are indicated by the horizontal red line. The prevalence of infection (% infected/total) is depicted for mosquitoes under each experimental condition and examined by X^2^ analysis to determine significance. Asterisks denote significance (*P < 0.05, **P < 0.01); ns, not significant. n = number of individual mosquitoes examined.

Since PGE2 signaling regulates the expression of multiple PPO and AMP genes (**Figures 2** and **S7**), we hypothesized that these immune molecules may directly mediate the effects of PGE2 signaling on oocyst survival. Previous studies have implicated PPOs (14) and AMPs (30, 31) in antagonizing *Plasmodium* survival, yet not every member of these respective gene families have been examined. Although DEF1 does not have a role in parasite infection (32), *PPO3* silencing has been demonstrated to produce a late-phase phenotype (14) similar to that of *Ag*PGE2R. However, *PPO-1, 7, 8* and *CEC1* have not previously been evaluated by RNAi for their role in anti-*Plasmodium* immunity. Following the injection of dsRNA for each gene of interest, each respective transcript was significantly reduced (**Figures 4A** and **S11**). However, only PPO1 significantly influenced oocyst survival at eight days post-infection (**Figures 4A** and **S11**). Based on the role of PPO1 (**Figure 4A**) and PPO3 (14) on parasite survival, and the influence of PGE2 signaling on their expression (**Figure 2**), we hypothesized that both PPO1 and PPO3 were responsible for the oocyst killing responses produced following PGE2 treatment. To validate this hypothesis, we co-silenced *PPO1* and *PPO3* (**Figure 4B**), and examined the effects of PGE2 treatment on *Plasmodium* oocyst numbers at eight days post-infection. In contrast to GFP-silenced controls displaying reduced oocyst numbers following PGE2 treatment (**Figure 4B**), PGE2 treatment had no effect in the *PPO1/3*-silenced background (**Figure 4B**), providing strong support that PPO1 and PPO3 mediate the oocyst killing responses produced by PGE2 signaling.

### PGE2 Triggers Oenocytoid Cell Lysis

**Figure 5:**
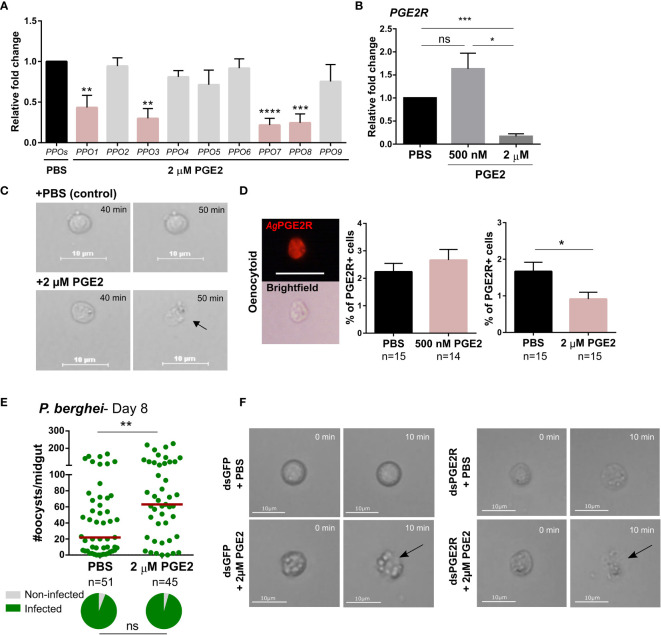
High concentration of PGE2 triggers oenocytoid lysis. **(A)** The influence of high PGE2 concentrations (2 µM) was examined on prophenoloxidase (PPO) gene expression, where the subset of PPO genes regulated by PGE2 treatment (*PPO1, 3, 7* and *8*) are significantly reduced. **(B)** Similarly, *AgPGE2R* expression was also reduced when mosquitoes were primed with 2 µM PGE2, yet was unaffected by 500 nM PGE2. and PBS control. **(C)** To determine whether high concentration of PGE2 triggers oenocytoid cell lysis, video recordings were performed for one hour, focusing on oenocytoids after exposure to 1xPBS or 2 µM PGE2. Still images of oenocytoids were captured at 40 min and 50 min of the incubation. Oenocytoid cell lysis (black arrow) was observed around 50 min incubation of 2 µM PGE2. To verify if oenocytoid immune cell populations were influenced by PGE2 levels, immunofluorescence assays (IFAs) were performed to label mosquito oenocytoids **(D)**. There was no difference in the proportion of *Ag*PGE2R+ oenocytoid cells between PBS and 500 nM PGE2 treatments, yet 2 µM PGE2 treatments reduced oenocytoid populations supporting that high levels of PGE2 promote oenocytoid lysis. The scale bar represents 10 µm. **(E)** When oenocytoid lysis was triggered by 2 µM PGE2 treatment prior to *P. berghei* infection, *Plasmodium* survival was significantly increased when oocyst numbers were examined 8 days post-infection. Similar to **(C)**, video recordings were performed focusing on oenocytoids after exposure to 1xPBS or 2 µM PGE2 in either GFP- or PGE2R-silenced mosquitoes **(F)**. Still images of oenocytoids were captured at 0 min and 10 min following incubation. Oenocytoid cell lysis (black arrow) was observed around 10 min incubation of 2 µM PGE2. Data presented in **(A, B, D)** were analyzed using an unpaired t-test to determine differences in relative gene expression or oenocytoid abundance using GraphPad Prism 7.0. Bars represent mean ± SE of three independent biological replicates for **(A, B)**, and two independent biological replicates for **(D)** Oocyst data from three independent experiments were analyzed by Mann–Whitney. Median oocyst numbers are indicated by the horizontal red line. The prevalence of infection (% infected/total) is depicted for mosquitoes under each experimental condition and examined by X^2^ analysis to determine significance. Asterisks denote significance (*P < 0.05, **P < 0.01, ***P < 0.001, ****P < 0.0001); ns, not significant. n = number of individual mosquitoes examined. Genes that display significant differences in gene expression following PGE2 treatment are shaded in pink.

In addition to the activation of oenocytoid-specific PPO and AMP transcripts, PGE2 treatment also increased PO activity (**Figure 2**). Based on studies in other insect systems, immune cell populations comparable to mosquito oenocytoids rupture shortly after immune activation (6, 18, 33–35), a process that involves prostaglandin signaling in lepidopteran insects (6, 18, 34). As a result, we investigated whether PGE2 might similarly induce oenocytoid lysis in *An. gambiae*. In contrast to the induction of *PPO* genes upregulated by PGE2 treatment at 500 nM (**Figure 2A**), higher concentrations of PGE2 (1 µM and 2 µM) decreased the expression of the same subset of PPO genes (PPO1/3/7/8) as compared to PBS controls (**Figures 5A** and **S7**). Moreover, relative *AgPGE2R* transcript was similarly reduced in mosquitoes following higher PGE2 concentrations (**Figure 5B**), while *AgPGE2R* remained unchanged following treatment with 500 nM of PGE2 (**Figure 5B**).

To more closely examine the potential that higher concentrations of PGE2 promote oenocytoid lysis, we treated perfused hemocytes with either 1xPBS or 2 µM of PGE2, following the fate of oenocytoids in liquid suspension on a hemocytometer after treatment. Cells treated with 1xPBS remained intact, while oenocytoids consistently ruptured ~40 minutes after treatment with 2 µM of PGE2 (**Figure 5C** and **Movies S1**, **S2**), providing clear evidence that oenocytoid cell lysis is mediated by PGE2. In contrast, PGE2 treatment did not result in granulocyte lysis (**Movies S3**, **S4**), indicating that the influence of PGE2 on cell rupture is specific to oenocytoids.

To further validate the effects of PGE2 on oenocytoid rupture, we examined 500 nM and 2 µM concentrations of PGE2 on oenocytoid populations using immunofluorescence assays (IFAs). We demonstrate that the administration of 500 nM of PGE2 has no effect on the proportion of *Ag*PGE2R^+^ cells, yet at higher concentrations of PGE2 (2 µM), the *Ag*PGE2R^+^ cells were significantly reduced (**Figure 5D**). Together, this suggests that lower levels of PGE2 promote immune activation in oenocytoid populations, that may eventually lead to cell rupture, while higher concentrations promote immediate cell rupture and the release of oenocytoid cell contents into the mosquito hemolymph. Supporting this theory, when mosquitoes are treated with 2 µM of PGE2 prior to *P. berghei* challenge, oocyst survival is significantly increased (**Figure 5E**). This is in direct contrast to infection outcomes following 500 nM of PGE2 treatment that make mosquitoes more resistant to infection (**Figure 3**), suggesting that intact oenocytoids are required to promote the effects of PGE2 priming and *P. berghei* oocyst killing.

Additional experiments were performed to examine oenocytoid lysis in *GFP*- and *AgPGE2R*-silenced mosquitoes to determine the role of *Ag*PGE2R in oenocytoid rupture. Similar to **Figure 5C**, PGE2 triggered oenocytoid cell rupture when administered to cells from *GFP*-silenced mosquitoes (**Figure 5F** and **Movies S5**, **S6**). However, PGE2 treatment also resulted in oenocytoid rupture in the *PGE2R*-silenced mosquitoes (**Figure 5F** and **Movies S7**, **S8**), suggesting that oenocytoid cell lysis *via* PGE2 is independent of *Ag*PGE2R, similar to studies in lepidopteran insects (6, 36). Of note, oenocytoid cell rupture occurred within 10 minutes in dsRNA-injected mosquitoes as opposed to ~50 minutes in naïve mosquitoes (**Figure 5C**), implying that previous injection/injury may sensitize oenocytoid populations.

## Discussion

The interactions between the innate immune system and malaria parasites are key determinants in shaping mosquito vector competence (29, 37). Therefore, significant effort has been devoted towards defining the immune molecules and immunological mechanisms that determine malaria parasite killing in the mosquito host. In other insect systems, prostaglandins have been implicated in insect cellular immune responses such as hemocyte spreading and chemotaxis, nodule formation, melanization, and encapsulation (10, 38, 39), yet have only been examined in limited studies in *An. gambiae* (11). In this study, we describe a PGE2 receptor (*Ag*PGE2R) in *An. gambiae*, exhibiting similarities to recently characterized insect PGE2 receptors in *M. sexta* (8) and *S. exigua* (10). With significant roles described for prostaglandins in the insect cellular immune response (6, 8, 11), our efforts herein are focused on describing the function of *AgPGE2R* in mosquito immune cell populations and its contributions to mosquito innate immunity.

Using immunofluorescence assays, we demonstrate that *Ag*PGE2R is predominantly expressed in the oenocytoid, a non-phagocytic immune cell sub-type traditionally associated with prophenoloxidase (PPO) production (40), and to a lesser extent in a subset of phagocytic cells at a lower signal intensity. This is in agreement with recent single-cell RNA-seq analysis of *An. gambiae* immune cell populations (22). However, our experiments demonstrate that *AgPGE2R* is unaffected by phagocyte depletion, suggesting that *AgPGE2R* expression in oenocytoids is responsible for the majority of its expression. These findings are also consistent with recent evidence that the *M. sexta* PGE2R is immunolocalized to oenocytoid immune cell populations (8), arguing that oenocytoids are central components of PGE2 signaling.

**Figure 6:**
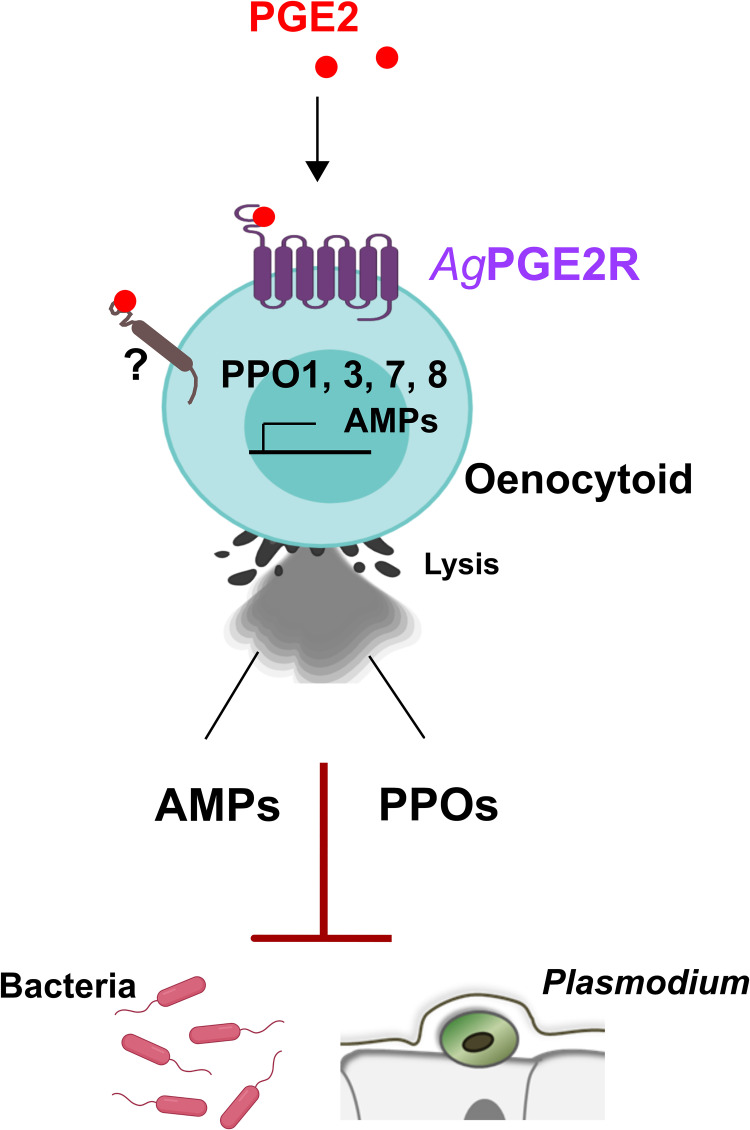
Summary of PGE2 signaling *via* PGE2R to activate *An. gambiae* immunity in oenocytoid immune cell populations. We demonstrate that PGE2 interacts with its cognate receptor, *Ag*PGE2R, to initiate innate immune expression of prophenoloxidases (PPOs) and AMPs in oenocytoid immune cell populations that contribute to bacteria and malaria parasite killing. PGE2 promotes oenocytoid cell lysis by PGE2, yet is independent of PGE2R through an unknown receptor.

Our data demonstrate that *Ag*PGE2R has an integral role in oenocytoid function, influencing the transcription of PPO and AMP genes, PO activity, and oenocytoid rupture. Moreover, these data provide definitive evidence for the role of oenocytoids in mosquito innate immunity, demonstrating an involvement in limiting bacteria and malaria parasite survival (summarized in **Figure 6**). However, we cannot discount other PGE2 signaling events that may influence the immune system. Oenocytoid rupture, and the release of its contents in the hemolymph, may also indirectly influence granulocytes or other components of humoral immunity to limit invading pathogens. With weak *AgPGE2R* expression in a subset of granulocytes, PGE2 treatment may directly or indirectly mediate hemocyte recruitment to the midgut basal lamina and promote granulocyte proliferation as previously described (11). Yet, despite these observed cellular immune responses, we cannot rule out the potential contributions of PGE2 signaling by other tissues expressing *AgPGE2R*. While our results suggest that *AgPGE2R* is expressed at lower levels in midgut and carcass tissues, other studies have suggested that *AgPGE2R* is not enriched in mosquito hemocyte populations (41, 42). As a result, PGE2 signaling in non-hemocyte tissues may also contribute additional humoral responses to bacterial and *Plasmodium* infection. However, at present, the systemic effects of *AgPGE2R*-silencing limit our ability to further address the tissue-specific contributions of PGE2 signaling in the mosquito innate immune response.

Focusing on the influence of *Ag*PGE2R in mosquito immune function, we demonstrate that PGE2 treatment influences PPO gene expression and subsequent downstream PO activity. Of interest, we found that PGE2 signaling influences the expression of a subset of PPO genes (*PPO1, -3, -7* and *-8*), which coincides with the same subset of PPO genes that are enriched in oenocytoid cell populations (22). Following PGE2 treatment, this subset of PPO genes was significantly upregulated, while *AgPGE2R*-silencing significantly reduced their expression, supporting the novel contributions of PGE2 signaling on a subset of PPOs. Recent studies have also demonstrated that the expression of *PPO1, -3*, and *-8* is mediated by the transcription factor lozenge (22), an integral regulator of oenocytoid (or comparable *Drosophila* crystal cell) differentiation (22, 43, 44), however our initial experiments suggest that *lozenge* expression is not directly influenced by PGE2 signaling. In addition, the regulation of a subset of PPOs *via* PGE2 signaling further highlight differences in gene regulation between PPO genes expressed in oenocytoids (*PPO1, -3, -7* and *-8*) and granulocytes *(PPO2*, -*4*, -*5*, -*6*, -*9)* (14, 22), providing support for distinct mechanisms of PPO regulation in mosquito immune cell populations that require further study.

Previous studies have demonstrated that injury and bacterial infection cause the release of PPO from *Drosophila* crystal cells (33), supporting that the increase in hemolymph PGE2 levels following *Plasmodium* infection may similarly trigger oenocytoid rupture. Through our experiments, we provide evidence that increasing PGE2 concentrations promote oenocytoid rupture in a concentration-dependent manner, similar to the role of prostaglandins in mediating the release of PPOs *via* oenocytoid rupture in the beet armyworm *S. exigua* (6, 18, 36). Comparable experiments using PGE2 concentrations that promote oenocytoid lysis, do not similarly promote granulocyte rupture, suggesting that these PGE2-medited responses are cell-type specific and not the result of general cellular toxicity produced due to high PGE2 levels. However, similar to other insect systems (6, 36), our data suggests that oenocytoid rupture is independent of *Ag*PGE2R function *via* yet undescribed mechanisms.

Our data also provide new insight into the role of PGE2 signaling in the mosquito “late-phase” immune response (14, 25–29). Similar to the phenotypes previously described for *PPO2*-, *PPO3*- and *PPO9*-silencing (14), we demonstrate that *PPO1*-silencing increases oocyst survival, further perpetuating the idea that the downstream components of PO activation promote *Plasmodium* oocyst killing. While the non-melanizing effects that contribute to these phenotypes have thus far proven elusive, the co-silencing of *PPO1* and *PPO3* eliminated the protective effects of PGE2 treatment, arguing that PPO expression is central to anti-*Plasmodium* effects of PGE2 signaling. This is further supported by our experiments using high PGE2 concentrations to deplete oenocytoid populations prior to *P. berghei* challenge, which importantly implicate oenocytoid function in the establishment of anti-*Plasmodium* immunity. Together with previous work (11, 14, 25, 26), these experiments support a model in which PGE2 is produced in the midgut following *Plasmodium* ookinete invasion (11), that contributes towards the cellular immune responses that mediate late-phase anti-*Plasmodium* immunity (14, 25, 26) and immune priming (11).

In addition to PPO regulation, we also provide evidence that PGE2 signaling stimulates the production of AMPs in *An. gambiae*, similar to previous studies in other systems (4, 13, 45). In light of this result, we demonstrate the role of PGE2 in mediating antibacterial immune responses that suppress bacterial growth, effects that are abrogated by *AgPGE2R*-silencing, indicating that *Ag*PGE2R is required for the antibacterial responses associated with PGE2 signaling.

In summary, our characterization of the PGE2 receptor in *An. gambiae* provides important new insights into the roles of PGE2 signaling in oenocytoid immune cell function and mosquito innate immunity. We demonstrate that *Ag*PGE2R mediates PPO and AMP gene expression that limit *Plasmodium* oocyst survival and suppress bacterial infection, establishing that PGE2 promotes oenocytoid rupture in *An. gambiae*. Together, this work reveals an integral role of oenocytoids in PGE2 signaling and mosquito innate immune function.

## Data Availability Statement

The original contributions presented in the study are included in the article/**Supplementary Material**. Further inquiries can be directed to the corresponding author.

## Ethics Statement

The animal study was reviewed and approved by Animal Care and Use Committee at Iowa State University.

## Author Contributions

Conceived and designed the experiments: HK and RS. Performed the experiments: HK and DH. Analyzed the data: HK, DH, and RS. Contributed reagents/materials/analysis tools: HK, DH, and RS. Wrote the paper: HK, DH, and RS. All authors contributed to the article and approved the submitted version.

## Funding

This work was supported by a Postdoctoral Association Seed Grant Award from Iowa State University (to HK) and R21 AI144705 from the National Institutes of Health, National Institute of Allergy and Infectious Diseases (to RS).

## Conflict of Interest

The authors declare that the research was conducted in the absence of any commercial or financial relationships that could be construed as a potential conflict of interest.

## Publisher’s Note

All claims expressed in this article are solely those of the authors and do not necessarily represent those of their affiliated organizations, or those of the publisher, the editors and the reviewers. Any product that may be evaluated in this article, or claim that may be made by its manufacturer, is not guaranteed or endorsed by the publisher.

## References

[1] Dennis EA Norris PC. Eicosanoid Storm in Infection and Inflammation. Nat Rev Immunol (2015) 15:511–23. 10.1038/nri3859 PMC4606863 26139350

[2] Moreno JJ. Eicosanoid Receptors: Targets for the Treatment of Disrupted Intestinal Epithelial Homeostasis. Eur J Pharmacol (2017) 796:7–19. 10.1016/j.ejphar.2016.12.004 27940058

[3] Woodward DF Jones RL Narumiya S. International Union of Basic and Clinical Pharmacology. LXXXIII: Classification of Prostanoid Receptors, Updating 15 Years of Progress. Pharmacol Rev (2011) 63:471–538. 10.1124/pr.110.003517 21752876

[4] García Gil de Muñoz FL Martínez-Barnetche J Lanz-Mendoza H Rodríguez MH Hernández-Hernández FC. Prostaglandin E2 Modulates the Expression of Antimicrobial Peptides in the Fat Body and Midgut of *Anopheles albimanus* . Arch Insect Biochem Physiol (2008) 68:14–25. 10.1002/arch.20232 18412259

[5] Ramos S Custódio A Silveira H. *Anopheles gambiae* Eicosanoids Modulate *Plasmodium berghei* Survival From Oocyst to Salivary Gland Invasion. Mem Inst Oswaldo Cruz (2014) 109:668–71. 10.1590/0074-0276140098 PMC4156460 25141285

[6] Shrestha S Stanley D Kim Y. PGE2induces Oenocytoid Cell Lysis Via a G Protein-Coupled Receptor in the Beet Armyworm, *Spodoptera exigua* . J Insect Physiol (2011) 57:1568–76. 10.1016/j.jinsphys.2011.08.010 21867708

[7] Merchant D Ertl RL Rennard SI Stanley DW Miller JS. Eicosanoids Mediate Insect Hemocyte Migration. J Insect Physiol (2008) 54:215–21. 10.1016/j.jinsphys.2007.09.004 17996890

[8] Kwon H Yang Y Kumar S Lee DW Bajracharya P Calkins TL. Characterization of the First Insect Prostaglandin (PGE2) Receptor: MansePGE2R Is Expressed in Oenocytoids and Lipoteichoic Acid (LTA) Increases Transcript Expression. Insect Biochem Mol Biol (2020) 117:103290. 10.1016/j.ibmb.2019.103290 31790798

[9] Kwon H Smith RC. Inhibitors of Eicosanoid Biosynthesis Reveal That Multiple Lipid Signaling Pathways Influence Malaria Parasite Survival in *Anopheles gambiae* . Insects (2019) 10:307. 10.3390/insects10100307 PMC6835628 31547026

[10] Kim Y Ahmed S Baki A Kumar S Kim K Park Y. Deletion Mutant of PGE2 Receptor Using CRISPR-Cas9 Exhibits Larval Immunosuppression and Adult Infertility in a Lepidopteran Insect, *Spodoptera exigua* . Dev Comp Immunol (2020) 111:103743. 10.1016/j.dci.2020.103743 32464135

[11] Barletta ABF Trisnadi N Ramirez JL Barillas-Mury C. Mosquito Midgut Prostaglandin Release Establishes Systemic Immune Priming. iScience (2019) 19:54–62. 10.1016/j.isci.2019.07.012 31351392 PMC6661395

[12] Ramirez JL de Almeida Oliveira G Calvo E Dalli J Colas RA Serhan CN. A Mosquito Lipoxin/Lipocalin Complex Mediates Innate Immune Priming in *Anopheles gambiae* . Nat Commun (2015) 6:7403. 10.1038/ncomms8403 26100162 PMC4542143

[13] Barletta ABF Alves E Silva TL Talyuli OAC Luna-Gomes T Sim S Angleró-Rodríguez Y. Prostaglandins Regulate Humoral Immune Responses in *Aedes aegypti* . PloS Negl Trop Dis (2020) 14:e0008706. 10.1371/journal.pntd.0008706 33095767 PMC7584201

[14] Kwon H Smith RC. Chemical Depletion of Phagocytic Immune Cells in *Anopheles gambiae* Reveals Dual Roles of Mosquito Hemocytes in Anti-*Plasmodium* Immunity. Proc Natl Acad Sci (2019) 116:14119–28. 10.1073/pnas.1900147116 PMC6628807 31235594

[15] Kumar S Stecher G Tamura K. MEGA7: Molecular Evolutionary Genetics Analysis Version 7.0 for Bigger Datasets. Mol Biol Evol (2016) 33:1870–4. 10.1093/molbev/msw054 PMC8210823 27004904

[16] Livak KJ Schmittgen TD. Analysis of Relative Gene Expression Data Using Real-Time Quantitative PCR and the 2(-Delta Delta C(T)) Method. Methods (2001) 25:402–8. 10.1006/meth.2001.1262 11846609

[17] Kumar JR Smith JP Kwon H Smith RC. Use of Clodronate Liposomes to Deplete Phagocytic Immune Cells in *Drosophila melanogaster* and *Aedes aegypti* . Front Cell Dev Biol (2021) 9:627976. 10.3389/fcell.2021.627976 33604338 PMC7884637

[18] Shrestha S Kim Y. Eicosanoids Mediate Prophenoloxidase Release From Oenocytoids in the Beet Armyworm Spodoptera exigua. Insect Biochem Mol Biol (2008) 38:99–112. 10.1016/j.ibmb.2007.09.013 18070669

[19] Kim IH Castillo JC Aryan A Martin-Martin I Nouzova M Noriega FG. A Mosquito Juvenile Hormone Binding Protein (mJHBP) Regulates the Activation of Innate Immune Defenses and Hemocyte Development. PloS Pathog (2020) 16:e1008288. 10.1371/journal.ppat.1008288 31961911 PMC6994123

[20] Zhang Z Austin SC Smyth EM. Glycosylation of the Human Prostacyclin Receptor: Role in Ligand Binding and Signal Transduction. Mol Pharmacol (2001) 60:480–7. 11502878

[21] Hirata T Narumiya S. Prostanoids as Regulators of Innate and Adaptive Immunity. Adv Immunol (2012) 116:143–74. 10.1016/B978-0-12-394300-2.00005-3 23063076

[22] Kwon H Mohammed M Franzén O Ankarklev J Smith RC. Single-Cell Analysis of Mosquito Hemocytes Identifies Signatures of Immune Cell Sub-Types and Cell Differentiation. eLife (2021) 10:e66192. 10.7554/eLife.66192 34318744 PMC8376254

[23] Midega J Blight J Lombardo F Povelones M Kafatos F Christophides GK. Discovery and Characterization of Two Nimrod Superfamily Members in *Anopheles gambiae* . Pathog Glob Health (2013) 107:463–74. 10.1179/204777213X13867543472674 PMC4073527 24428830

[24] Estévez-Lao TY Hillyer JF. Involvement of the *Anopheles gambiae* Nimrod Gene Family in Mosquito Immune Responses. Insect Biochem Mol Biol (2014) 44:12–22. 10.1016/j.ibmb.2013.10.008 24200842

[25] Smith RC Barillas-Mury C Jacobs-Lorena M. Hemocyte Differentiation Mediates the Mosquito Late-Phase Immune Response Against *Plasmodium* in *Anopheles gambiae* . Proc Natl Acad Sci (2015) 112:E3412–20. 10.1073/pnas.1420078112 PMC4491748 26080400

[26] Kwon H Arends BR Smith RC. Late-Phase Immune Responses Limiting Oocyst Survival Are Independent of TEP1 Function Yet Display Strain Specific Differences in *Anopheles gambiae* . Parasit Vectors (2017) 10:369. 10.1186/s13071-017-2308-0 28764765 PMC5540282

[27] Gupta L Molina-Cruz A Kumar S Rodrigues J Dixit R Zamora RE. The STAT Pathway Mediates Late-Phase Immunity Against *Plasmodium* in the Mosquito *Anopheles gambiae* . Cell Host Microbe (2009) 5:498–507. 10.1016/j.chom.2009.04.003 19454353 PMC2701194

[28] Goulielmaki E Siden-Kiamos I Loukeris TG. Functional Characterization of *Anopheles* Matrix Metalloprotease 1 Reveals Its Agonistic Role During Sporogonic Development of Malaria Parasites. Infect Immun (2014) 82:4865–77. 10.1128/IAI.02080-14 PMC4249346 25183733

[29] Smith RC Barillas-Mury C. Plasmodium Oocysts: Overlooked Targets of Mosquito Immunity. Trends Parasitol (2016) 32:979–90. 10.1016/j.pt.2016.08.012 27639778

[30] Reynolds RA Kwon H Smith RC. 20-Hydroxyecdysone Primes Innate Immune Responses That Limit Bacterial and Malarial Parasite Survival in *Anopheles gambiae* . mSphere (2020) 5:e00983–19. 10.1128/mSphere.00983-19 PMC7160685 32295874

[31] Kokoza V Ahmed A Woon Shin S Okafor N Zou Z Raikhel AS. Blocking of *Plasmodium* Transmission by Cooperative Action of Cecropin A and Defensin A in Transgenic *Aedes aegypti* Mosquitoes. Proc Natl Acad Sci USA (2010) 107:8111–6. 10.1073/pnas.1003056107 PMC2889521 20385844

[32] Blandin S Moita LF Köcher T Wilm M Kafatos FC Levashina EA. Reverse Genetics in the Mosquito *Anopheles gambiae*: Targeted Disruption of the Defensin Gene. EMBO Rep (2002) 3:852–6. 10.1093/embo-reports/kvf180 PMC1084233 12189180

[33] Bidla G Dushay MS Theopold U. Crystal Cell Rupture After Injury in *Drosophila* Requires the JNK Pathway, Small GTPases and the TNF Homolog Eiger. J Cell Sci (2007) 120:1209–15. 10.1242/jcs.03420 17356067

[34] Shrestha S Kim Y. Oenocytoid Cell Lysis to Release Prophenoloxidase Is Induced by Eicosanoid via Protein Kinase C. J Asia Pac Entomol (2009) 12:301–5. 10.1016/j.aspen.2009.08.001

[35] Binggeli O Neyen C Poidevin M Lemaitre B. Prophenoloxidase Activation is Required for Survival to Microbial Infections in *Drosophila* . PloS Pathog (2014) 10:e1004067. 10.1371/journal.ppat.1004067 24788090 PMC4006879

[36] Shrestha S Park J Ahn SJ Kim Y. PGE2 Mediates Oenocytoid Cell Lysis Via a Sodium-Potassium-Chloride Cotransporter. Arch Insect Biochem Physiol (2015) 89:218–29. 10.1002/arch.21238 25845372

[37] Smith RC Vega-Rodríguez J Jacobs-Lorena M. The *Plasmodium* Bottleneck: Malaria Parasite Losses in the Mosquito Vector. Mem Inst Oswaldo Cruz (2014) 109:644–61. 10.1590/0074-0276130597 PMC4156458 25185005

[38] Stanley D Kim Y. Prostaglandins and Their Receptors in Insect Biology. Front Endocrinol (2011) 2:105. 10.3389/fendo.2011.00105 PMC3356066 22654840

[39] Kim Y Ahmed S Stanley D An C. Eicosanoid-Mediated Immunity in Insects. Dev Comp Immunol (2018) 83:130–43. 10.1016/j.dci.2017.12.005 29225005

[40] Hillyer JF Strand MR. Mosquito Hemocyte-Mediated Immune Responses. Curr Opin Insect Sci (2014) 3:14–21. 10.1016/j.cois.2014.07.002 25309850 PMC4190037

[41] Pinto SB Lombardo F Koutsos AC Waterhouse RM McKay K An C. Discovery of *Plasmodium* Modulators by Genome-Wide Analysis of Circulating Hemocytes in *Anopheles gambiae* . Proc Natl Acad Sci USA (2009) 106:21270–5. 10.1073/pnas.0909463106 PMC2783009 19940242

[42] Raddi G Barletta AB Efremova M Ramirez JL Cantera R Teichmann S. Mosquito Cellular Immunity at Single-Cell Resolution. Science (2020) 369:1128–32. 10.1126/science.abc0322 PMC8405044 32855340

[43] Fossett N Hyman K Gajewski K Orkin SH Schulz RA. Combinatorial Interactions of Serpent, Lozenge, and U-Shaped Regulate Crystal Cell Lineage Commitment During *Drosophila* Hematopoiesis. Proc Natl Acad Sci U.S.A. (2003) 100:11451–6. 10.1073/pnas.1635050100 PMC208778 14504400

[44] Waltzer L Ferjoux G Bataillé L Haenlin M. Cooperation Between the GATA and RUNX Factors Serpent and Lozenge During *Drosophila* Hematopoiesis. EMBO J (2003) 22:6516–25. 10.1093/emboj/cdg622 PMC291817 14657024

[45] Bernard JJ Gallo RL. Cyclooxygenase-2 Enhances Antimicrobial Peptide Expression and Killing of *Staphylococcus Aureus* . J Immunol (2010) 185:6535–44. 10.4049/jimmunol.1002009 PMC3025174 20971925

