# Supplementary material for: Prostaglandin E2 Signaling Mediates Oenocytoid Immune Cell Function and Lysis, Limiting Bacteria and Plasmodium Oocyst Survival in Anopheles gambiae
Source: Front Immunol. 2021 Aug 16;12:680020. doi: 10.3389/fimmu.2021.680020 (PMC8415482; doi:10.3389/fimmu.2021.680020)
Supplement: Supplementary file 1 [file DataSheet_1.pdf]

Supplemental Information

Prostaglandin E2 signaling mediates oenocytoid immune cell function and lysis, limiting bacteria and *Plasmodium* oocyst survival in *Anopheles gambiae*

Hyeogsun Kwon, David R. Hall and Ryan C. Smith*

Department of Entomology, Iowa State University, Ames, Iowa 50011, USA

* Corresponding author: smithr@iastate.edu

Supplemental Information

Supplemental Figures

Figure S1. Cloning of the *Ag*PGE2 receptor (*Ag*PGE2R) and amino acid translation.

Figure S2. Predicted secondary structure and phylogenetic analysis of the *An. gambiae* prostaglandin E2 receptor (AgPGE2R).

Figure S3. Relative abundance of *AgPGE2R* transcript in mosquito tissues under different physiological conditions.

Figure S4. *Ag*PGE2R localization to mosquito immune cells.

Figure S5. Expression of *AgPGE2R* and PPOs in mosquito immune cell populations.

Figure S6. Analysis of mosquito PGE2 titers under different physiological conditions.

Figure S7. Relative gene expression of PPOs following PGE2 treatment.

Figure S8. Efficiency of *AgPGE2R*-silencing.

Figure S9. PGE2 signaling does not significantly influence *lozenge* expression.

Figure S10. PGE2 signaling promotes antimicrobial activity.

Figure S11. Evaluation of other PGE2-regulated genes by RNAi that do not result in oocyst phenotypes.

Supplemental Tables

Table S1. Primers for cloning, qRT-PCR, and dsRNA synthesis.

Supplemental Movies

Movie S1. A perfused oenocytoid with the addition of 1xPBS.

Movie S2. A perfused oenocytoid with the addition of 2 µm PGE2.

Movie S3. Perfused granulocytes with the addition of 1xPBS.

Movie S4. Perfused granulocytes with the addition of 2 µm PGE2.

Movie S5. A perfused oenocytoid from *GFP*-silenced mosquitoes with the addition of 1xPBS.

Movie S6. A perfused oenocytoid from *GFP*-silenced mosquitoes with the addition of 2 µm PGE2.

Movie S7. A perfused oenocytoid from *PGE2R*-silenced mosquitoes with the addition of 1xPBS.

Movie S8. A perfused oenocytoid from *PGE2R*-silenced mosquitoes with the addition of 2 µm PGE2.

```
      GTGCGGTTGCACGAGCTAACGCGAGCTGAATCATCCCCAAAGGGGTATTTGATAGA   -192
CAAAGTTGAAGACGGTGTAGTCTTTAAGTTGTCTCGAGTGTGTGTGTACGTGTGCGTGTTACTGCA   -127
AACAAATCAGCATCATGAACTTTCCCGTGCAATAATAAGGTCGAATATACGAAGTACCAACAAGTG   -61
AAGTGTACTCCGCCAACGATCAAACGTGCGAATACTTCCCTGAACGTAAAGGCATTGGTCATGGCA   6
                                                            M  A     2
TCGCACAGTTTCCTTCCTGGAGTGTCCGTGAACGGGACGGTTCTGACCAACTATACACAGCTTGCT   66
 S  H  S  F  L  P  G  V  S  V  N  G  T  V  L  T  N  Y  T  Q  L  A    24

GCTGCTGCCGGCGCCCTGCTCACCACCATCAGCAATGGTACGGAGGCCGGCGAGCTGGCGGCCAGC   132
 A  A  A  G  A  L  L  T  T  I  S  N  G  T  E  A  G  E  L  A  A  S    46

AATCGGACGCAACCAAAGCCTGGACCCTCGCTGATGACGCCCTGCATGGTGATAGTGATGCTTTCG   198
 N  R  T  Q  P  K  P  G  P  S  L  M  T  P  C  M  V  I  V  M  L  S    68

TACATTTTCGGCTGCGTTGGGAACCTGATGGCCCTCATCCATCTGTGGCGCAACGTGCGGAACACG   264
 Y  I  F  G  C  V  G  N  L  M  A  L  I  H  L  W  R  N  V  R  N  T    90

AAGCATGCGCTAATGCTGAAATGTTTACTCACGAACGATCTCATCGGCTTGAGCGGCATGTTTGTC   330
 K  H  A  L  M  L  K  C  L  L  T  N  D  L  I  G  L  S  G  M  F  V    112

CAGATGTGTTTGCATCTTTACCTTTCGCCCGACGTGGTGCAGGCGAACATTCACAACCTTTGCGTC   396
 Q  M  C  L  H  L  Y  L  S  P  D  V  V  Q  A  N  I  H  N  L  C  V    134

CTGCGCGTAATTTGGCGTGTATTCGGCATCAGTTCCGGCTGCGTCGCGTTCGTCATGGCCCTGGAA   462
 L  R  V  I  W  R  V  F  G  I  S  S  G  C  V  A  F  V  M  A  L  E    156

CGCTACATAGCGCTGGCGAAACCGTTCTTTTATCATAAGTACGTGGCGAATAAGCTGATCCGCAAA   528
 R  Y  I  A  L  A  K  P  F  F  Y  H  K  Y  V  A  N  K  L  I  R  K    178

TCGATCTTTATCCTGTGGGGCATCGGAGCGTTCATAACGTTTCTGCCCCTGCTTGGGTTCGGTGTG   594
 S  I  F  I  L  W  G  I  G  A  F  I  T  F  L  P  L  L  G  F  G  V    200

TACTTTGACGAACGGAAGCAAACGTGTGTCCGGTACCGGAGCGCCACCGAACCGATCGACGTGGCG   660
 Y  F  D  E  R  K  Q  T  C  V  R  Y  R  S  A  T  E  P  I  D  V  A    222

TACGCATATTTGTTCTTCGCTGTTGGAACGTTGCTGTGCGTGGGGATCGTGATCTGTAATCTCAGC   726
 Y  A  Y  L  F  F  A  V  G  T  L  L  C  V  G  I  V  I  C  N  L  S    244

GTAAGGAAGGTGCTGTACCAATCGCACCGCAAGATGTGCCGCCAGTTTGGTTCGATCAAACCGACC   792
 V  R  K  V  L  Y  Q  S  H  R  K  M  C  R  Q  F  G  S  I  K  P  T    266

CCCATGCTGAACCGCTCCATGAGCCAAACGCCCAAATCGTCTAGCTTTACCGACTCCAACATTATA   858
 P  M  L  N  R  S  M  S  Q  T  P  K  S  S  S  F  T  D  S  N  I  I    288

CGTATGTTTAACGAGCCCACGACGGAGGAAATACGCTTCGCCAAGCTGATGACCGTTCTCAGTGTG   924
 R  M  F  N  E  P  T  T  E  E  I  R  F  A  K  L  M  T  V  L  S  V    310

TTCTTCATCATCTGCTGGCTTCCACAGATGATCTCAATCATCTTGTTGCAACAGCTCAGCGCTGCC   990
 F  F  I  I  C  W  L  P  Q  M  I  S  I  I  L  L  Q  Q  L  S  A  A    332

ATGAAGCTGAAGCTATCCTGGGTGTTCCGCGTGTCGGACATACTGATACTGGTGCACTTTATGCTC   1056
 M  K  L  K  L  S  W  V  F  R  V  S  D  I  L  I  L  V  H  F  M  L    354

GATCCGTACATCTACGTGCTGCTGAAGAAGAGCCGCCGCAGCGATCTGCGCACCATGATACGCTAC   1122
 D  P  Y  I  Y  V  L  L  K  K  S  R  R  S  D  L  R  T  M  I  R  Y    376

ATGTTTAGTCGCAACCAGCGGTTCAACATGGTCGATGTCGCCCTATCACCGATGCAGAAATCGACC   1188
 M  F  S  R  N  Q  R  F  N  M  V  D  V  A  L  S  P  M  Q  K  S  T    398

AACTCATCGCCACTTCCGTAG                                                1209
 N  S  S  P  L  P  *                                                 404
```

Figure S1. Cloning of the *Ag*PGE2 receptor (*Ag*PGE2R) and amino acid translation. cDNA prepared from perfused hemocytes of female adult naïve *An. gambiae* revealed a 1463 bp sequence, encoding a 404 amino acid residue protein. Seven transmembrane

regions are underlined (●——●), while highly conserved residues in Family A GPCRs (E156, R157, Y158) are shaded. Using the MyHits online analysis tool, N-linked glycosylation sites (single-underlined) were predicted in the N-terminus. Residues (double-underlined) in the C-terminus indicates prediction of potential phosphorylation sites by protein-kinase A and C.

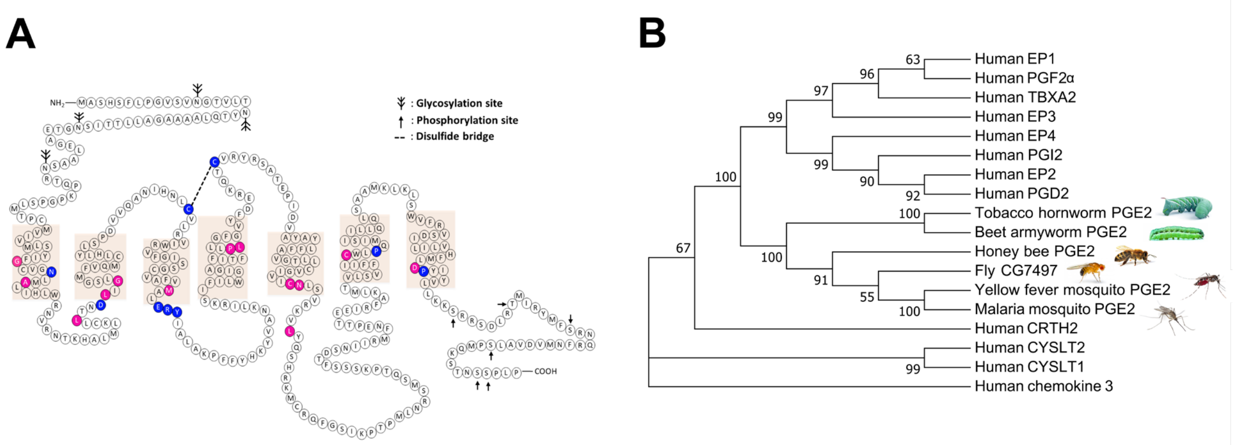

Figure S2. Predicted secondary structure and phylogenetic analysis of the *An. gambiae* prostaglandin E2 receptor (*Ag*PGE2R). *Ag*PGE2R amino acid residues circled in blue are conserved in the rhodopsin-like family (Family A) of GPCRs, while residues circled in magenta are conserved with human prostanoid receptors (**A**). The seven transmembrane domains predicted by TMpred are displayed by beige-shaded boxes. Four N-glycosylated sites are predicted at the N-terminus and phosphorylation sites are located at the C-terminus. A single palmitoylation site was predicted by the presence of three cysteines at the C-terminus. (**B**) Phylogenetic analysis was performed with human prostanoid receptors, human leukotriene receptors, and putative insect PGE2 receptors (PGE2Rs). Human chemokine receptor 3 was used as outgroup to root the tree. Neighbor-joining method with 1000 bootstrap replicates was used to construct the tree using MEGA 7.0. The following GenBank accession number indicates different amino acid sequence found insects. Tobacco hornworm (*Manduca sexta*) PGE2R (QDO79407.1), beet armyworm (*Spodoptera exigua*) PGE2R (QEN91980.1), honey bee (*Apis mellifera*) PGE2R (XP_001120030.2), fruit fly (*Drosophila melanogaster*) (NP_648999.2), yellow fever mosquito (*Aedes aegypti*) PGE2R (XP_021705923.1), malaria mosquito (*Anopheles gambiae*) PGE2R (XP_321552.2). The NCBI accession numbers of human eicosanoid and chemokinin receptors are listed below. Human EP1 (AAP32302.1: prostaglandin E receptor 1 subtype EP1), Human PGF2α (NP_000950.1: prostaglandin F2-alpha receptor isoform a precursor), Human TBXA2, (NP_963998.2: thromboxane A2 receptor isoform beta), Human EP3 (AAA60076.1: prostaglandin receptor EP3A1). Human EP4 (NP_000949.1: prostaglandin E2 receptor EP4 subtype), Human PGI2 (NP_000951.1: prostacyclin receptor), Human EP2 (NP_000947.2: prostaglandin E2 receptor EP2 subtype), Human PGD2 (NP_000944.1: prostaglandin D2 receptor isoform 1), Human CRTH2 (NP_004769.2: prostaglandin D2 receptor 2), Human CYSLT2 (NP_065110.1: cysteinyl leukotriene receptor 2), Human CYSLT1 (NP_006630.1: cysteinyl leukotriene receptor 1), Human chemokine (NP_001495.1: C-X-C chemokine receptor type 3 isoform 1).

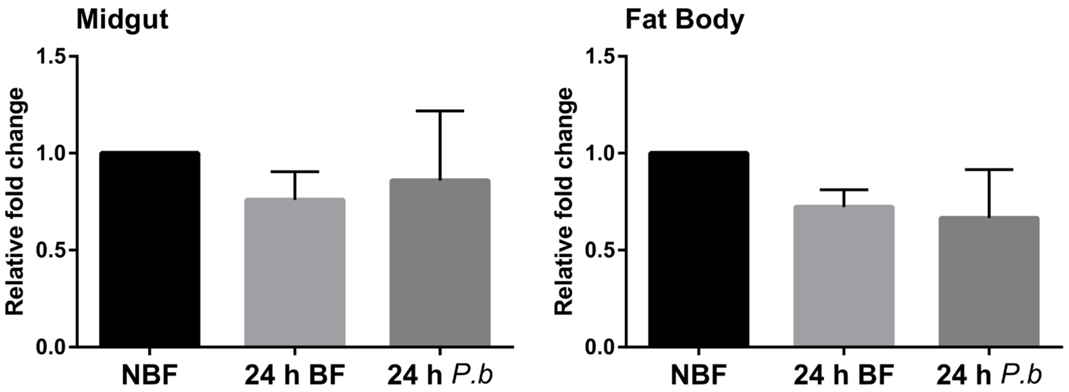

Figure S3. Relative abundance of *AgPGE2R* transcript in mosquito tissues under different physiological conditions. *AgPGE2R* expression was examined by qRT-PCR in midgut and fat body under naïve (NBF), blood-fed (24 h BF), or *P. berghei*-infected (24 h *P.b*) conditions. When analyzed by a one-way ANOVA followed by a Tukey's multiple comparison test using GraphPad Prism 7.0, no differences in expression were detected across physiological conditions. Bars represent the mean ± SE of either three or four independent biological replicates.

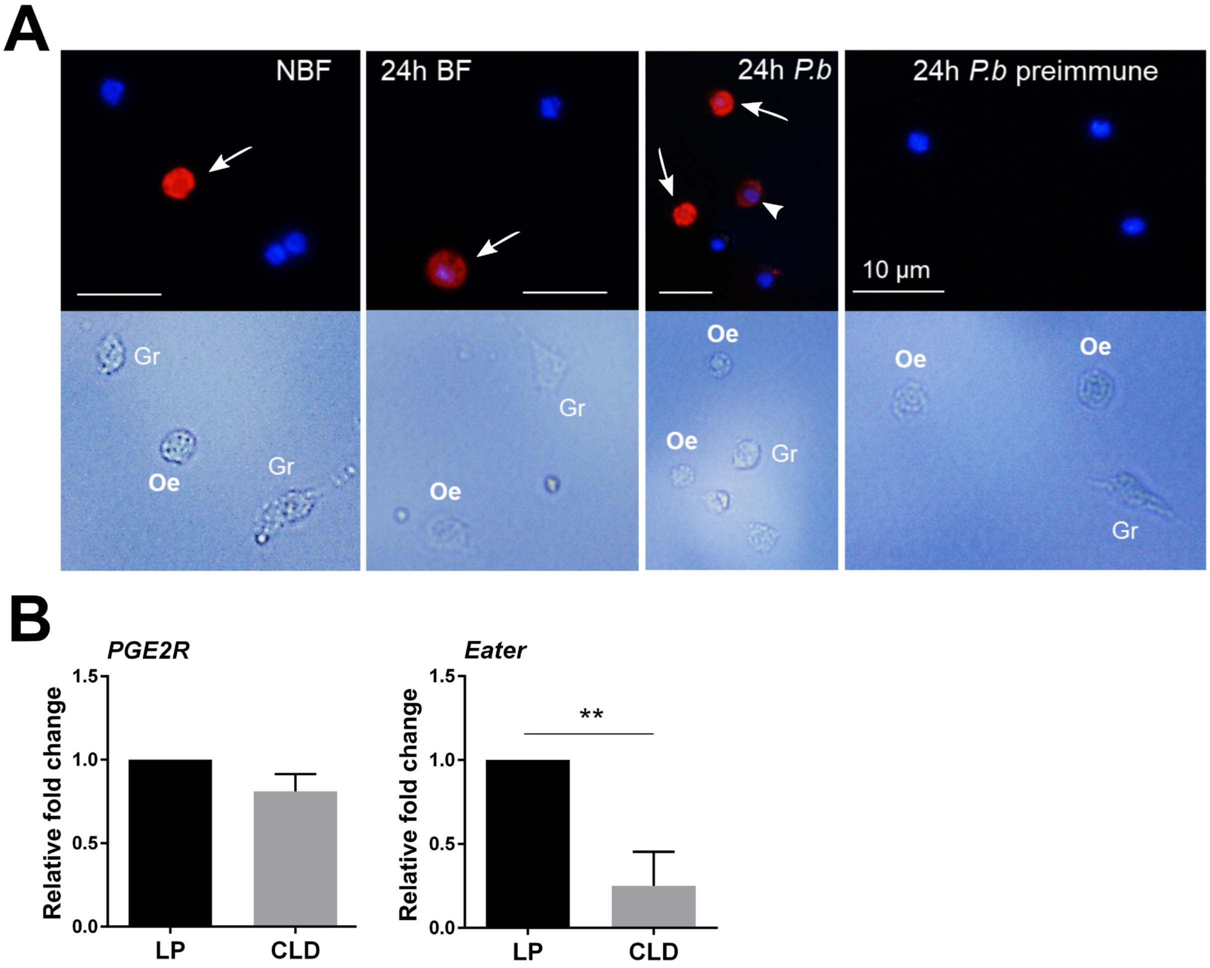

Figure S4. *Ag*PGE2R localization to mosquito immune cells. Immunofluorescence assays using an *Ag*PGE2R antibody recognizing extracellular loop 2 (ECL2) reveal that *Ag*PGE2R is predominantly expressed in oenocytoids (Oe, white arrows), although weak signal was detected in a subset of presumed granulocyte populations (Gr, white arrowhead in 24 h *P.b*) (**A**). Scale bar represents 10 μm. (**B**) To validate that receptor signal is derived from the non-phagocytic oenocytoids, mosquitoes were treated with clodronate liposomes (CLD) to deplete phagocytic granulocytes. Empty liposomes (LP) were used as a control. qRT-PCR analysis demonstrates that *AgPGE2R* transcript is not reduced following CLD treatment, while the expression of the phagocyte marker *eater* was significantly reduced, supporting *AgPGE2R* is expressed primarily in the oenocytoids. Data were analyzed by an unpaired t using GraphPad Prism 7.0. Bars represent mean ± SE of three independent replications. Asterisks denote significance (**$P < 0.01$).

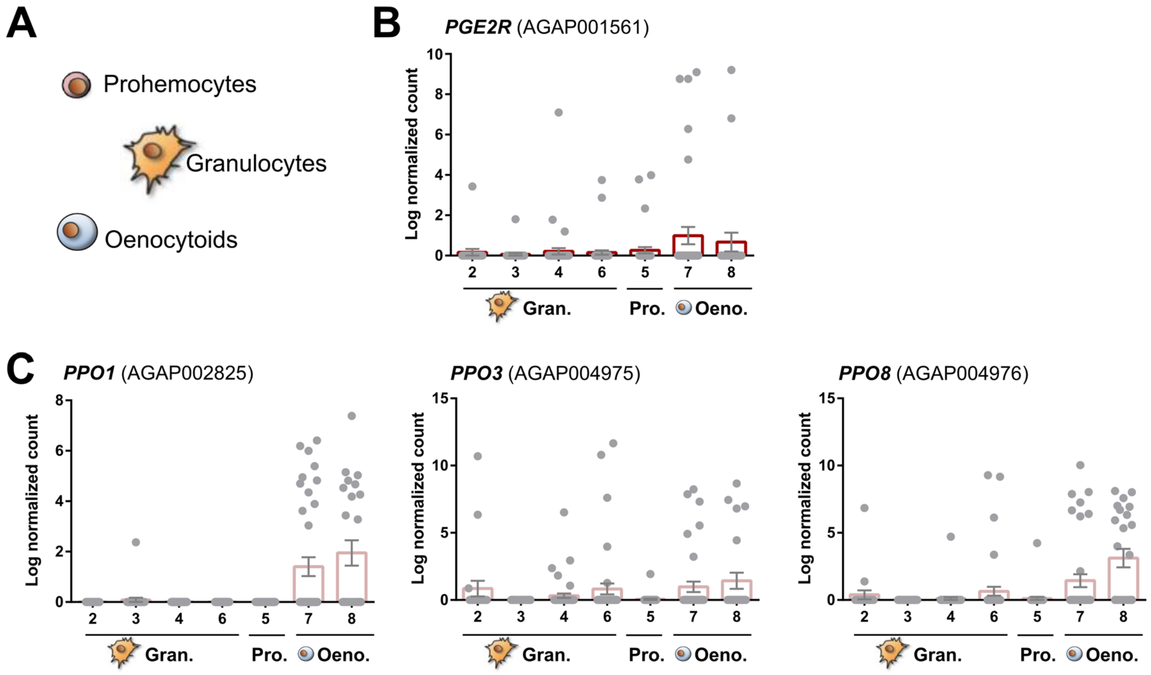

Figure S5. **Expression of *Ag*PGE2R and PPOs in mosquito immune cell populations.** Mosquito immune cells (hemocytes) can be classified into three primary cell types (**A**), with additional sub-types within the granulocyte or oenocytoid lineages. Using recent single-cell data from Kwon *et al.* (Kwon *et al.*, 2020- *bioRxiv*), the expression of *AgPGE2R* (**B**) or *PPO1*, *PPO3*, and *PPO8* (**C**) in each of the defined immune cell clusters (numbers) belonging to granulocyte, prohemocyte, or oenocytoid cell types. Grey dots correspond to gene expression (log normalized count) from an individual cell belonging to each respective cell type. Columns display the mean ± SE for each respective cell cluster.

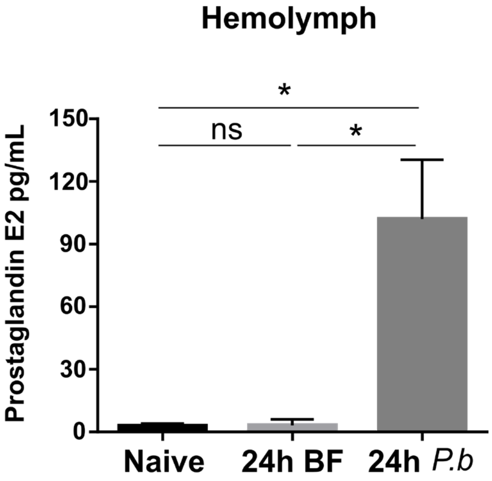

Figure S6. Analysis of mosquito PGE2 titers under different physiological conditions. PGE2 titers were measured from perfused hemolymph under naïve, at 24 h blood feeding (24 h BF), or 24h *P. berghei* infection (24 h *P.b*) conditions. Hemolymph samples were collected from 10 mosquitoes per treatment. Data were analyzed using a one-way ANOVA followed by Tukey post-test using GraphPad Prism 7.0. Bars represents mean ± SE of three independent biological replicates. Asterisks denote significance (*$P < 0.05$); ns, not significant.

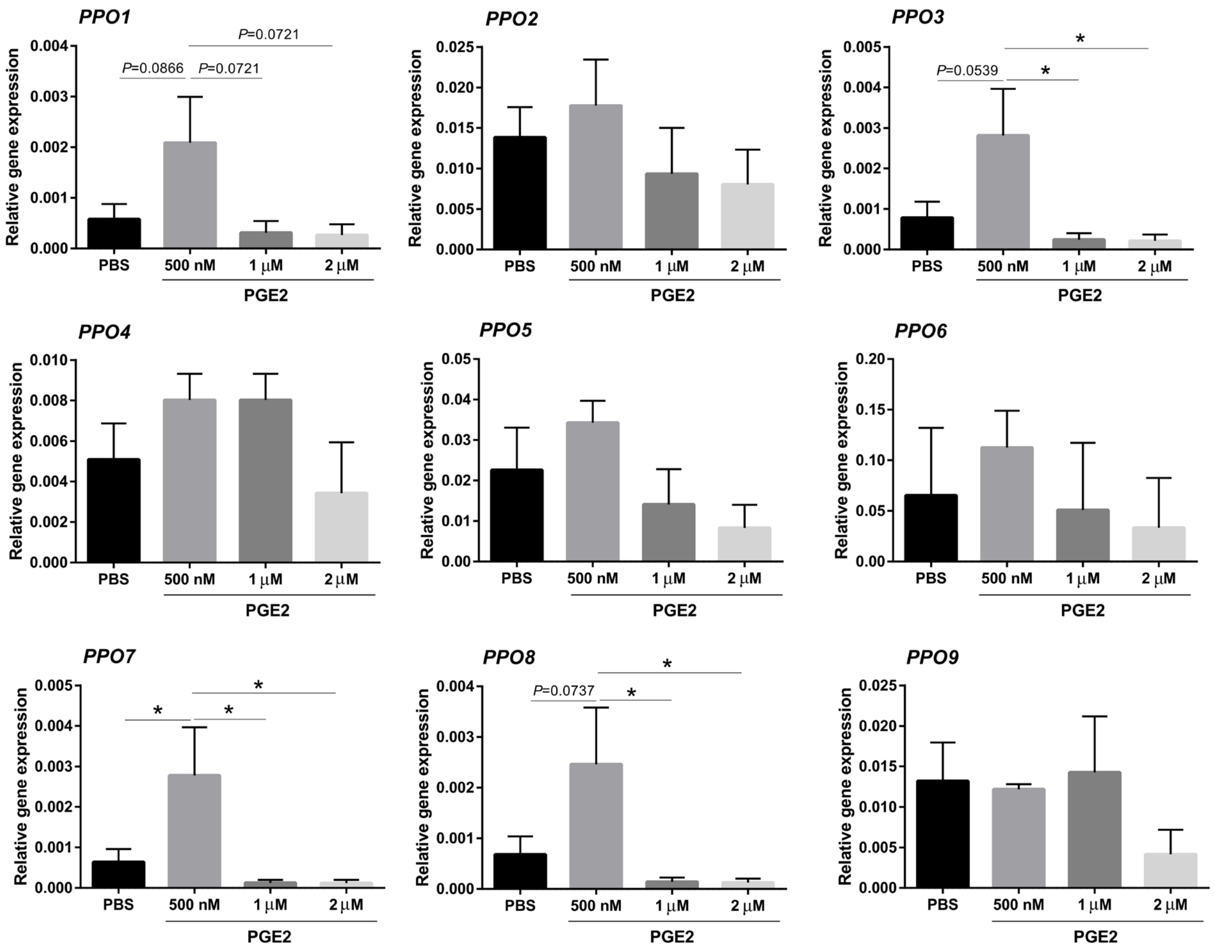

Figure S7. Relative gene expression of PPOs following PGE2 treatment. Each of the nine PPO family members were examined by qRT-PCR following the injection of 1xPBS or PGE2 at various concentrations (500 nM, 1 μM, or 2 μM). For each PPO gene, expression levels are displayed relative to expression levels of *rpS7*. Data were analyzed by a one-way ANOVA using a Holm-Sidak's multiple comparison test to determine differences in relative gene expression for each respective PPO gene between treatments using GraphPad Prism 7.0. Bars represent mean ± SE of three independent biological replicates. Asterisks denote significance (**P* < 0.05).

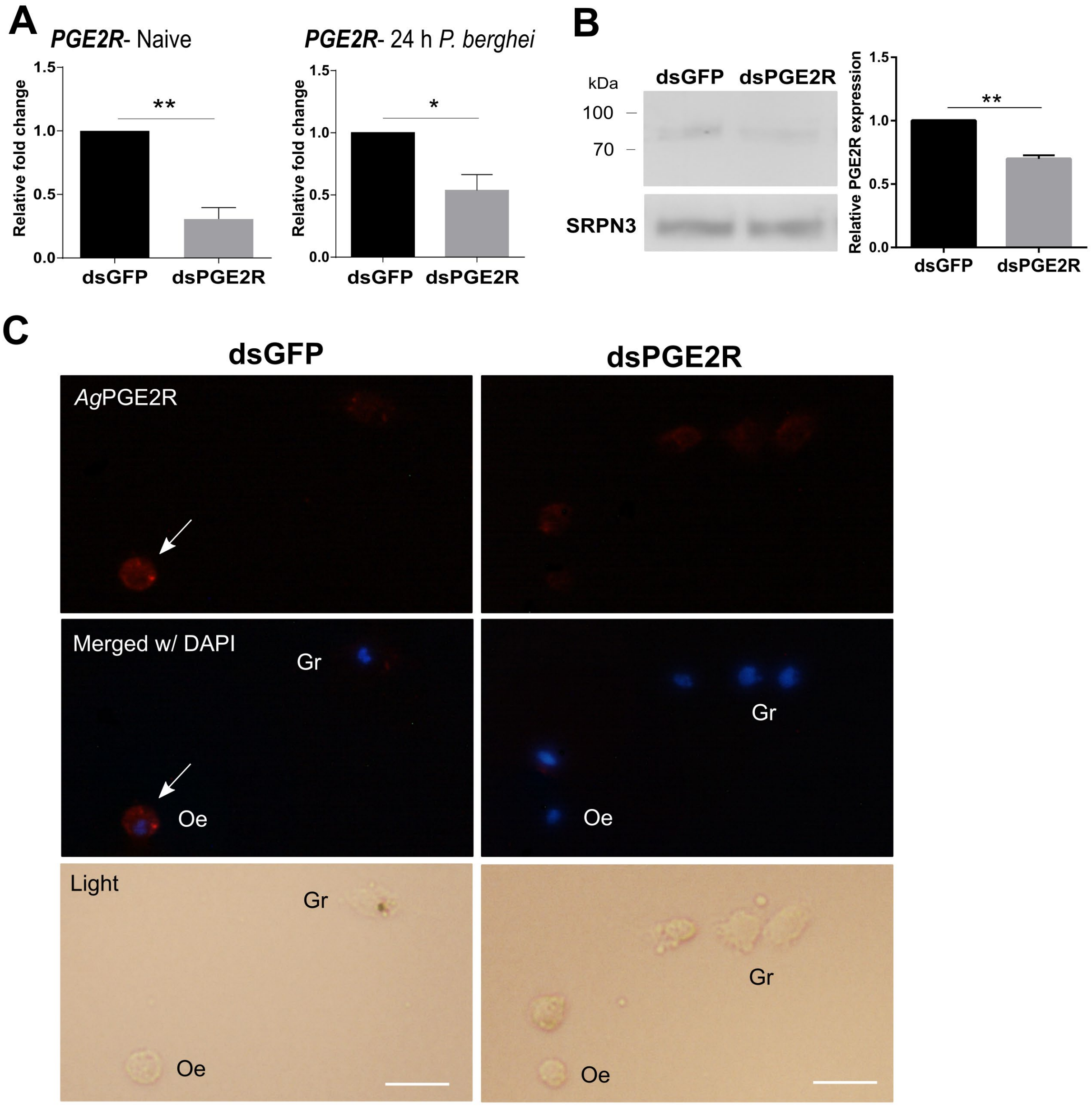

Figure S8. Efficiency of *AgPGE2R*-silencing. *AgPGE2R* expression was examined by qRT-PCR analysis (**A**), Western blot (**B**) and immunofluorescent assay (**C**) analyses to validate the efficacy of RNAi. Whole mosquitoes were examined from naïve and 24 h P. berghei-infected samples by qRT-PCR (**A**). Hemolymph perfused from naïve mosquitoes at day 4 post-injection of dsRNA was used for analysis of PGE2R protein expression. A serine protease inhibitor, serpin3 (SRPN3) was used as a protein loading control (**B**). To compare protein abundance between *GFP*- and *PGE2R*-silenced samples, densitometry was performed using Image J on independent experiments to display a significant knockdown of AgPGE2R protein expression (**B**). AgPGE2R was detected by

immunofluorescence in oenocytoids (Oe; white arrow), but not in the granulocytes (Gr) perfused from GFP control mosquitoes (**C**). AgPGE2R signal was reduced in immunofluorescence assays in *AgPGE2R*-silenced mosquitoes (**C**). qRT-PCR and densitometry data were analyzed using an unpaired t test to determine differences in relative gene expression or protein concentration using GraphPad Prism 7.0. Bars represent mean ± SE of three independent replications. Asterisks denote significance (**P* < 0.05, ***P* < 0.01). Scale bar represents 10 μm (**C**).

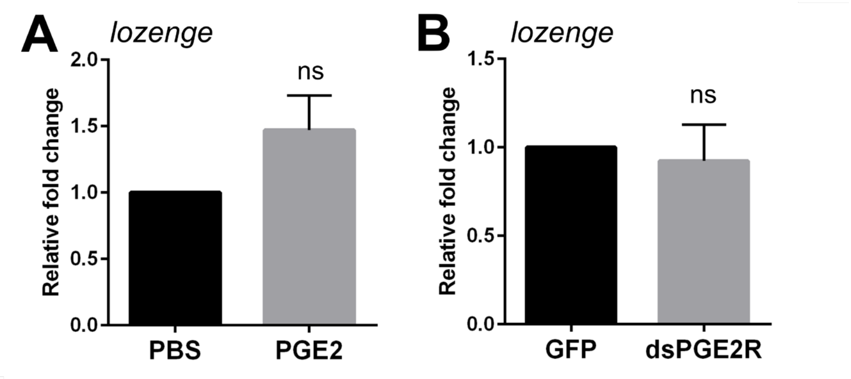

Figure S9. PGE2 signaling does not significantly influence *lozenge* expression. The expression of *lozenge* was examined by qRT-PCR analysis following 500 nM PGE2 treatment (**A**) or in *AgPGE2R*-silenced mosquitoes (**B**). Data were analyzed from three independent experiments using an unpaired t test to determine differences gene expression using GraphPad Prism 7.0. Bars represent the mean ± SE. ns, not significant.

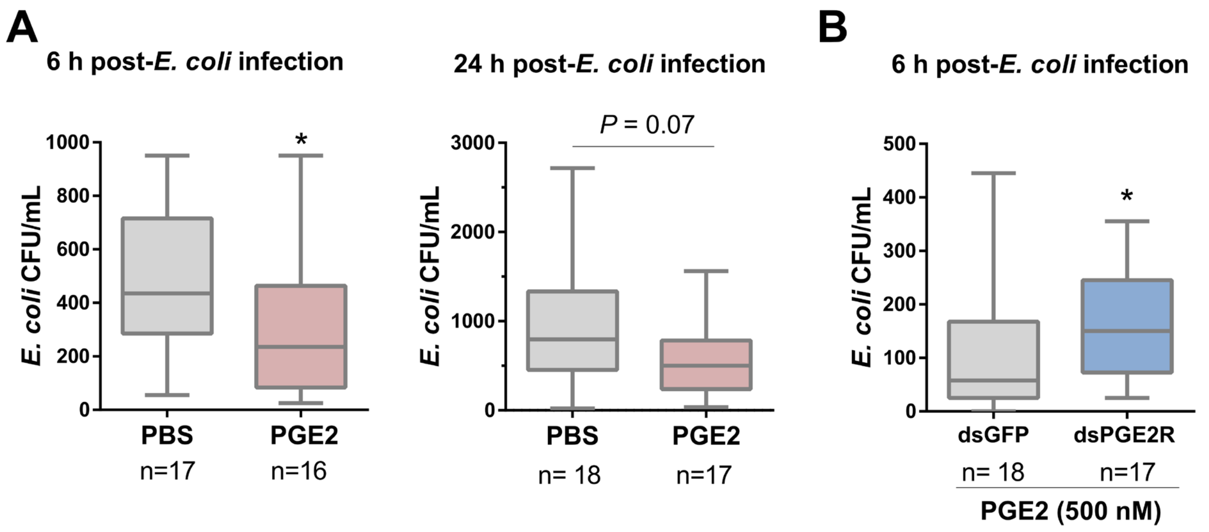

Figure S10. PGE2 signaling promotes antimicrobial activity. Naïve mosquitoes injected with 1x PBS or primed with PGE2 (500 nM) were challenged with *E. coli*, then bacteria titers were examined at 6 and 24 h post-infection (**A**). To validate that the antimicrobial activity of PGE2 occurred through *Ag*PGE2R, mosquitoes were silenced with dsGFP or dsPGE2R, primed with PGE2, then challenged with *E. coli* (**B**). *AgPGE2R*-silencing significantly impaired bacterial clearance compared to GFP control when evaluated 6 h post-infection. Data displaying the median values from three independent biological replicates are depicted as box and whisker plots (min and max) and analyzed by a Mann–Whitney test using GraphPad Prism 7.0. Asterisks denote significance (*$P <$ 0.05). n=number of individual mosquitoes examined.

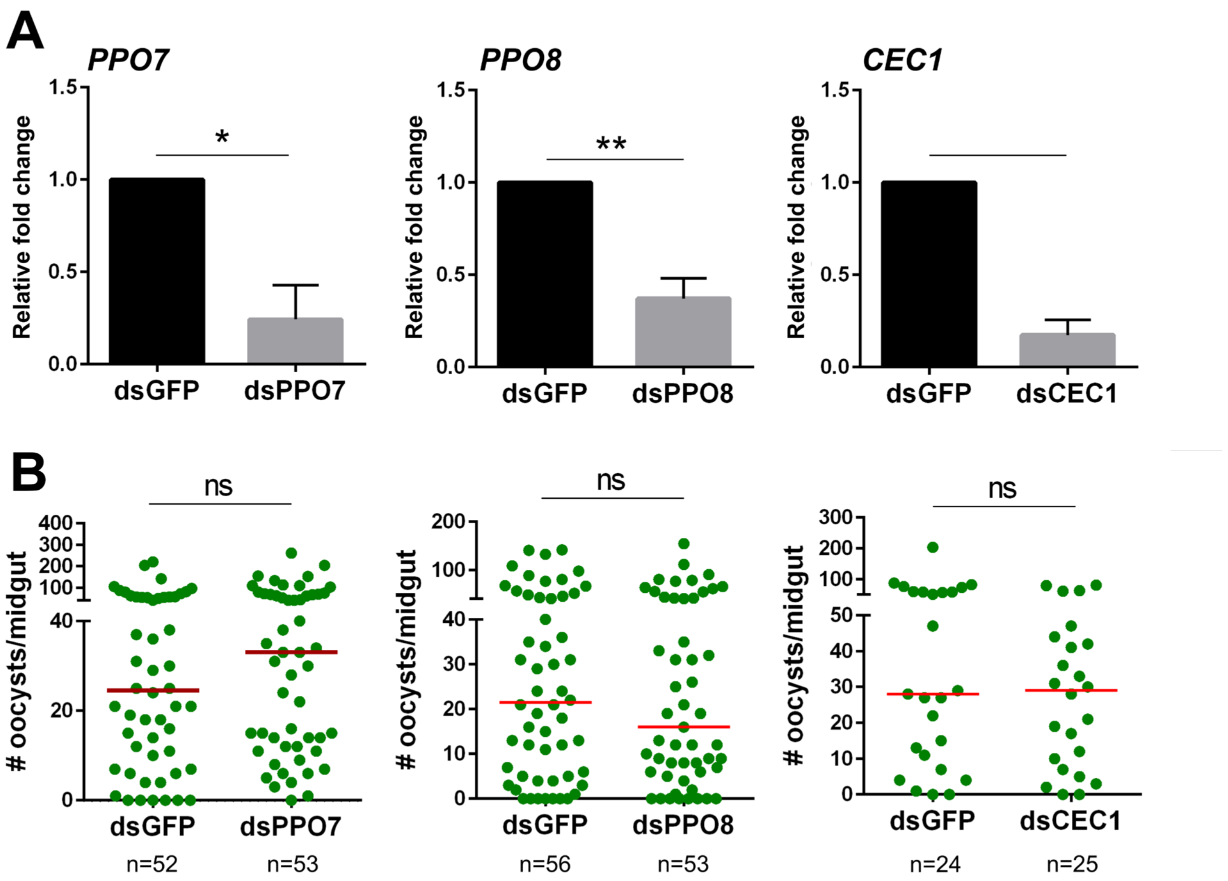

Figure S11. Evaluation of other PGE2-regulated genes by RNAi that do not result in oocyst phenotypes. The effects of dsRNA-mediated gene-silencing were evaluated in whole mosquitoes by qRT-PCR for *PPO7*, *PPO8*, and *CEC1* as compared to GFP controls (**A**). Data were analyzed by an unpaired t-test using GraphPad Prism 6.0. Bars represent mean ± SE of three independent biological replicates. (**B**) Additional experiments were performed for each knockdown to evaluate its contributions to malaria parasite survival. None of the examined genes influenced parasite survival when oocysts numbers were examined 7 days post-infection. PPO7- and PPO8-silenced mosquitoes were examined in three independent biological experiments, while CEC1-silenced were examined from two independent biological experiments. Data were analyzed by a Mann–Whitney test to assess oocyst survival. Median oocyst numbers are indicated by the horizontal red line. Asterisks denote significance (*$P < 0.05$, **$P < 0.01$, ****$P < 0.0001$); ns, not significant. n=number of individual mosquitoes examined.

Table S1. Primers for cloning, qRT-PCR and dsRNA synthesis

Primer	Sequence (5’-3’)	Gene ID
**For cloning**		
pge2R-F	GTGCGGTTGCACGAGCTAAC	AGAP001561
pge2R-R	CTACGGAAGTGGCGATGAGTTGG	
**For qRT-PCR**		
pge2R-qF	GCTTCGCCAAGCTGATGACC	AGAP001561
pge2R-qR	ACGCGGAACACCCAGGATAG	
ppo1-qF	GACTCTACCCGGATCGGAAG	AGAP002825
ppo1-qR	ACTACCGTGATCGACTGGAC	
ppo2-qF	TTGCGATGGTGACCGATTTC	AGAP006258
ppo2-qR	CGACGGTCCGGATACTTCTT	
ppo3-qF	CTATTCGCCATGATCTCCAACTACG	AGAP004975
ppo3-qR	ATGACAGTGTTGGTGAAACGGATCT	
ppo4-qF	GCTACATACACGATCCGGACAACTC	AGAP004981
ppo4-qR	CCACATCGTTAAATGCTAGCTCCTG	
ppo5-qF	GTTCTCCTGTCGCTATCCGA	AGAP012616
ppo5-qR	CATTCGTCGCTTGAGCGTAT	
ppo6-qF	GCAGCGGTCACAGATTGATT	AGAP004977
ppo6-qR	GCTCCGGTAGTGTTGTTCAC	
ppo7-qF	CAGCGATTGACGAAGGTGTT	AGAP004980
ppo7-qR	GAAAGCAATACGTGCCCACT	
ppo8-qF	CCTTTGGTAACGTGGAGCAG	AGAP004976
ppo8-qR	CTTCAAACCGCGAGACCATT	
ppo9-qF	TGTATCCATCTCGGACGCAA	AGAP004978
ppo9-qR	AAGGTTGCCAACACGTTACC	
cec1-qF	CAGCAGAGAAGGCCCTACCG	AGAP000693
cec1-qR	TCATGTTAGCAGAGCCGTCGT	
cec4-qF	CCACGCTGCTACTGTTCGGT	AGAP006722
cec4-qR	CTGCAGTACGGGCACTACCT	
def1-qF	ATGCCGCGCTGGAGAACTAT	AGAP011294
def1-qR	ATAGCGGCGAGCGATACAGT	
Lz-qF	GCACCGTCAATCAGAACCAA	AGAP002506
Lz-qR	TGCCACTGATCGAATGCTTG	
rps7-qF	ACCACCATCGAACACAAAGTTGACACT	AGAP010592

rps7-qR	CTCCGATCTTTCACATTCCAGTAGCAC	
**For dsRNA synthesis**		
pge2R-T7F	TAATACGACTCACTATAGGGGTGCGGTTGCACGAGCTAAC	AGAP001561
pge2R-T7R	TAATACGACTCACTATAGGGAGGGCGTCATCAGCGAGGGT	
ppo1-T7F	TAATACGACTCACTATAGGGCATCGGTTCCGAAATCCAG	AGAP002825
ppo1-T7R	TAATACGACTCACTATAGGGCAGATCGAGATCGTGCGTAT	
ppo7-T7F	TAATACGACTCACTATAGGGGTACGAGTGCGGACACACC	AGAP004980
ppo7-T7R	TAATACGACTCACTATAGGGGAATTTGAGGAAACGCTGCT	
ppo8-T7F	TAATACGACTCACTATAGGGAAAATTTGGCCAATCGCTTT	AGAP004976
ppo8-T7R	TAATACGACTCACTATAGGGAACGAGTCCCGGCGTTTAAT	
cec1-T7F	TAATACGACTCACTATAGGGAGAGACCAACCAACCACCAA	AGAP000693
cec1-T7R	TAATACGACTCACTATAGGGTTAGCAGAGCCGTCGTCTTA	
GFP-T7F	TTAATACGACTCACTATAGGGAGAATGGTGAGCAAGGGCGAGGAGCTGT	
GFP-T7R	TTAATACGACTCACTATAGGGAGATTACTTGTACAGCTCGTCCATGCC	
